# Thymine DNA glycosylase as a novel target for melanoma

**DOI:** 10.1038/s41388-018-0640-2

**Published:** 2019-01-23

**Authors:** Pietro Mancuso, Rossella Tricarico, Vikram Bhattacharjee, Laura Cosentino, Yuwaraj Kadariya, Jaroslav Jelinek, Emmanuelle Nicolas, Margret Einarson, Neil Beeharry, Karthik Devarajan, Richard A. Katz, Dorjbal G. Dorjsuren, Hongmao Sun, Anton Simeonov, Antonio Giordano, Joseph R. Testa, Guillaume Davidson, Irwin Davidson, Lionel Larue, Robert W. Sobol, Timothy J. Yen, Alfonso Bellacosa

**Affiliations:** 1 Cancer Epigenetics Program, Fox Chase Cancer Center, 333 Cottman Avenue, Philadelphia, PA 19111 USA; 2 Department of Medical Biotechnologies, Universita’ degli Studi di Siena, Siena, Italy; 3 Institut Curie, PSL Research University, INSERM U1021, Normal and Pathological Development of Melanocytes, 91405 Orsay, France; 4 Cancer Biology Program, Fox Chase Cancer Center, 333 Cottman Avenue, Philadelphia, PA 19111 USA; 5 Fels Institute for Cancer and Molecular Biology, Temple University School of Medicine, Philadelphia, PA 19140 USA; 6 Department of Biostatistics, Fox Chase Cancer Center, 333 Cottman Avenue, Philadelphia, PA 19111 USA; 7 Division of Preclinical Innovation, National Center for Advancing Translational Sciences, National Institutes of Health, Rockville, MD 20850 USA; 8 Sbarro Institute for Cancer Research and Molecular Medicine, Department of Biology, College of Science and Technology, Temple University, Philadelphia, PA 19122 USA; 9 Institut de Génétique et de Biologie Moléculaire et Cellulaire, CNRS/INSERM/ULP, 67404 Illkirch, France; 10 Equipe Labellisée Ligue Contre le Cancer, Orsay, France; 11 University Paris-Sud, University Paris-Saclay, CNRS UMR3347, Orsay, France; 12 Department of Oncologic Sciences, Mitchell Cancer Institute, University of South Alabama, Mobile, AL 36604 USA

**Keywords:** Melanoma, Target identification

## Abstract

Melanoma is an aggressive neoplasm with increasing incidence that is classified by the NCI as a recalcitrant cancer, i.e., a cancer with poor prognosis, lacking progress in diagnosis and treatment. In addition to conventional therapy, melanoma treatment is currently based on targeting the BRAF/MEK/ERK signaling pathway and immune checkpoints. As drug resistance remains a major obstacle to treatment success, advanced therapeutic approaches based on novel targets are still urgently needed. We reasoned that the base excision repair enzyme thymine DNA glycosylase (TDG) could be such a target for its dual role in safeguarding the genome and the epigenome, by performing the last of the multiple steps in DNA demethylation. Here we show that *TDG* knockdown in melanoma cell lines causes cell cycle arrest, senescence, and death by mitotic alterations; alters the transcriptome and methylome; and impairs xenograft tumor formation. Importantly, untransformed melanocytes are minimally affected by *TDG* knockdown, and adult mice with conditional knockout of *Tdg* are viable. Candidate TDG inhibitors, identified through a high-throughput fluorescence-based screen, reduced viability and clonogenic capacity of melanoma cell lines and increased cellular levels of 5-carboxylcytosine, the last intermediate in DNA demethylation, indicating successful on-target activity. These findings suggest that TDG may provide critical functions specific to cancer cells that make it a highly suitable anti-melanoma drug target. By potentially disrupting both DNA repair and the epigenetic state, targeting TDG may represent a completely new approach to melanoma therapy.

## Introduction

Melanoma is an aggressive cancer, whose incidence has increased over the past two decades in Western countries [1]. Although the majority of melanoma cases are cured after surgical excision of the primary tumor, the metastatic form of the disease has poor prognosis, being highly resistant to therapy. Targeted therapy directed against *BRAF* is effective but short-lived, because resistance develops rapidly. More recently, immunotherapy based on checkpoint inhibition demonstrated responses in ~60% of advanced melanoma patients, but a large fraction of patients is refractory. Thus advanced therapeutic strategies based on novel targets are urgently needed.

We recently identified the requirement of the base excision repair enzyme thymine DNA glycosylase (TDG) for mammalian development and specifically for development of the neural crest, precursor of melanocytes [2]. This requirement is due to the unique dual role of TDG in safeguarding genome and epigenome [3, 4]. TDG not only protects CpG sites from spontaneous deamination of 5-methylcytosine (5mC) and cytosine, thus avoiding C>T transition mutations, but importantly, at the epigenomic level, is involved in active DNA demethylation downstream of the ten-eleven translocation (TET) dioxygenases [2–6].

Active DNA demethylation involves the iterative oxidation of 5mC by TET1–3 to produce the novel cytosine species 5-hydroxymethylcytosine (5hmC), 5-formylcytosine (5fC), and 5-carboxylxytosine (5caC), followed by TDG-mediated removal of 5fC and 5caC [7, 8]. In this pathway, isocitrate dehydrogenase (IDH) generates α-ketoglutarate, a cofactor for TET-mediated oxidation.

Alterations of DNA demethylation, through mutations/reduced expression of *IDH2* and *TET* family genes, have been described in melanoma and correlated with worse prognosis [9–15]. Moreover, decreased levels of 5hmC have been reported in melanoma and represent a novel epigenetic biomarker with diagnostic/prognostic implications [16, 17].

Given the importance of DNA demethylation in melanomagenesis and TDG requirement for neural crest development [2], we began exploring the role of TDG in melanoma. We reasoned that the two non-redundant (genomic and epigenomic) functions of TDG may represent a vulnerability of tumor cells that can be exploited as novel targets for treatment, because targeting TDG may have the double effect of altering DNA repair capacity and epigenetic state. In this study, through cell culture and mouse xenograft studies, we establish the importance of TDG in maintaining the viability of melanoma cells, and using a DNA repair molecular beacon assay [18], we isolate first-generation TDG inhibitors and characterize their anticancer activity.

## Results

### *TDG* is expressed in melanoma, and its knockdown induces morphological changes in melanoma cell lines

**Fig. 1:**
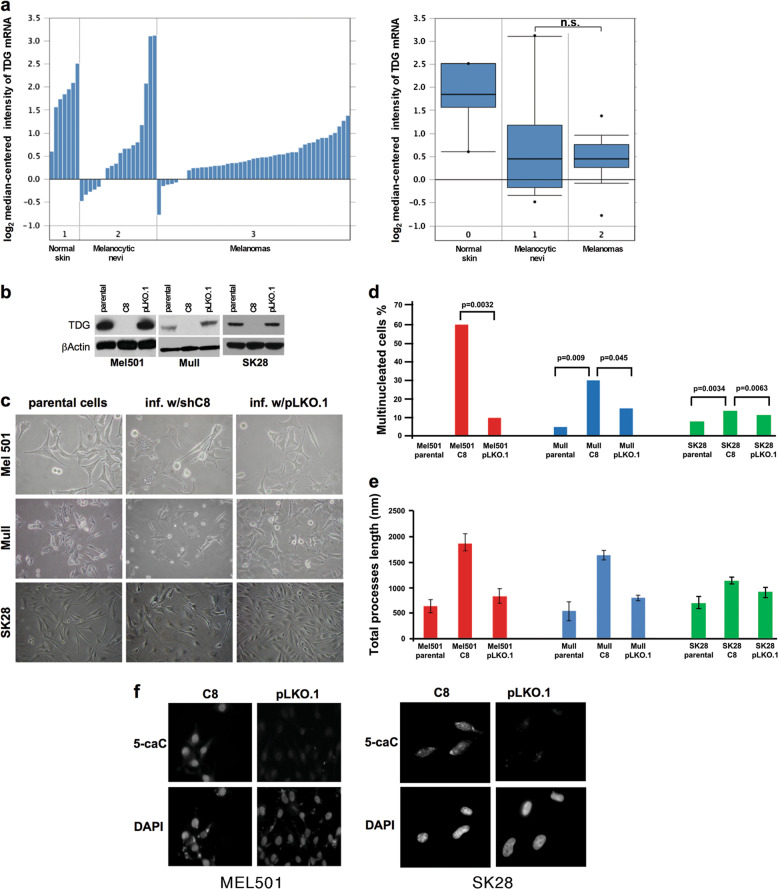
*TDG* knockdown induces morphological changes in melanoma cells. **a** Expression levels of *TDG* mRNA in normal human skin, melanocytic nevi, and cutaneous melanoma in Talantov melanoma dataset (Oncomine); both individual and boxplot representations are shown. The difference between the median TDG expression in melanocytic nevi and melanoma samples is not significant (n.s.) by Wilcoxon rank-sum test. **b** Western blot showing effective *TDG* knockdown in Mel501, Mull, and SK28 cells in comparison to parental and control pLKO.1-infected cells 3 days after infection. **c** Phase-contrast images of parental, *TDG* knockdown (C8), and control pLKO.1-infected Mel501, Mull, and SK28 cells. **d** Percentage of multinucleated cells and **e** total length of cellular processes in parental, *TDG* knockdown (C8), and control pLKO.1-infected Mel501, Mull, and SK28 cells. Data are presented as average ± standard deviation (SD). **f** Immunofluorescence staining for 5-carboxylcytosine (5caC) in *TDG* knockdown and control pLKO.1-infected Mel501 and SK28 cells. Nuclei are counter-stained with DAPI. P parental cells, C8 short hairpin RNA (shRNA) lentivirus against *TDG*, pLKO.1 lentivirus vector control

Examination of the Oncomine database (http://www.oncomine.org) revealed that the median expression levels of *TDG* mRNA are similar in melanoma samples and melanocytic nevi (levels are higher in normal skin, in which, however, the melanocytes are a minority) (Fig. 1a). In the Human Protein Atlas database, nuclear expression of TDG protein is also maintained at high-to-medium levels in melanomas; and high expression is associated with unfavorable prognosis (Suppl. Figure 1a; http://www.proteinatlas.org). Interestingly, in TCGA-SKCM (skin cutaneous melanoma) cases, there is a positive correlation between *TDG* and *TET1-3* mRNA expression levels (Suppl. Figure 1b). These observations were consistent with the possibility that TDG is a melanoma target and prompted us to examine the consequences of its knockdown.

TDG protein and mRNA are expressed in a panel of melanoma lines, at levels varying from low to high, and apparently inversely correlating with tumorigenic potential (Suppl. Figure 2). TDG mRNA and protein expression is also maintained in normal melanocyte cultures at levels similar to melanoma cell lines (Suppl. Figure 2) [19]. We used the C8 lentivirus expressing an short hairpin RNA (shRNA) against TDG [2] to knock down the TDG expression in Mel501, Mull, and SK28 melanoma lines (Fig. 1b). *TDG* knockdown induced marked morphological changes in all three cell lines (large, flat, multinucleated cells bearing numerous and long dendritic processes) (Fig. 1c–e and Suppl. Figure 3). Similar morphological changes were also observed in other melanoma cell lines (Rosi and MNT-1, Suppl. Figure 4a). Similar results were obtained with a second *TDG* shRNA lentivirus, sh4575, targeting a different region of *TDG* (Suppl. Figure 4b-c), which rules out off-target effects. The on-target knockdown efficiency of C8 and sh4575 were confirmed not only by the reduction of TDG protein levels but also by the expected increase of 5caC levels (Fig. 1f and Suppl. Figure 4d).

### *TDG* knockdown reduces proliferation and induces cell cycle arrest and multinucleation

**Fig. 2:**
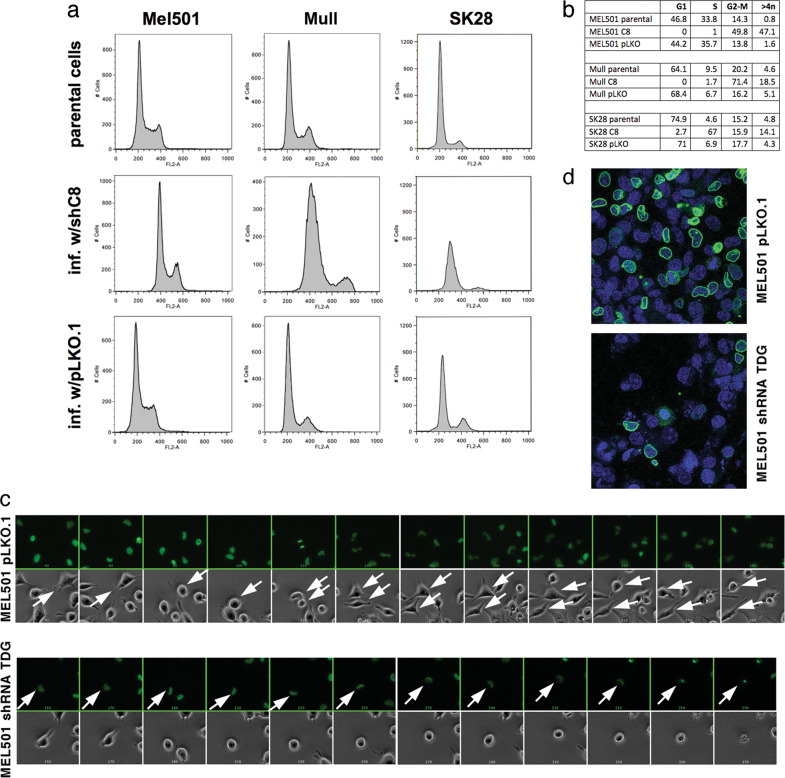
*TDG* knockdown in melanoma cells induces cell cycle arrest and causes mitotic alterations. **a** Fluorescence-activated cell sorting (FACS) of propidium iodide-stained parental, *TDG* knockdown, and control pLKO.1-infected Mel501, Mull, and SK28 cells showing that *TDG* knockdown induces cell cycle arrest in G2–M phase and S phase, respectively. Cell cycles were analyzed in biological triplicate 4 days after lentivirus infection. **b** Quantitation of cells in each phase of the cycle is shown. **c** Representative images of live cell videomicroscopy of GFP-tagged histone H2B-expressing MEL501 cells infected with shRNA against TDG and pLKO.1 control lentiviruses and progressing through mitosis. For each time point, bright field (top) and fluorescent (bottom) channels are shown. The time difference between adjacent frames is 300’. Arrows mark cells being tracked; two close arrows signify successful mitosis and two daughter cells. **d** Immunofluorescence detection of lamin B (green) in parental, *TDG* knockdown, and control pLKO.1-infected Mel501 cells. Nuclei were counter-stained with DAPI

Morphological changes were associated with decreased proliferation of Mel501, Mull, and SK28 melanoma lines. To elucidate the mechanism by which *TDG* knockdown reduces cell proliferation, we analyzed the cell cycle profile of parental, shC8-, and pLKO-infected Mel501, Mull, and SK28 cells by fluorescence-activated cell sorting (FACS). *TDG* knockdown perturbed cell cycle dynamics, causing a G2–M arrest in Mel501 and Mull cells and S-phase arrest in SK28 cells (Fig. 2a, b). Importantly, some cells with *TDG* knockdown escaped the G2–M or S-phase arrest, and accumulated >4*n* DNA content in agreement with the appearance of multinucleated cells (Fig. 1c).

To better understand the cell cycle arrest associated with *TDG* knockdown, we used time-lapse videomicroscopy to track the fates of individual Mel501 cells stably expressing histone H2B-green fluorescent protein (GFP) after infection with the pLKO or C8 lentivirus. Cells infected with C8 lentivirus failed to enter mitosis (Fig. 2c). This, along with the cell cycle profile (Fig. 2a, b) and 4,6-diamidino-2-phenylindole (DAPI) staining that confirmed the lack of mitotic cells (Fig. 2d), strongly suggests that the TDG-depleted MEL501 cells are delayed in G2. At later times in the movie, the C8-infected cells exhibited membrane blebbing and pyknotic nuclei, which was followed by cell death (Fig. 2c).

### *TDG* knockdown also induces senescence

**Fig. 3:**
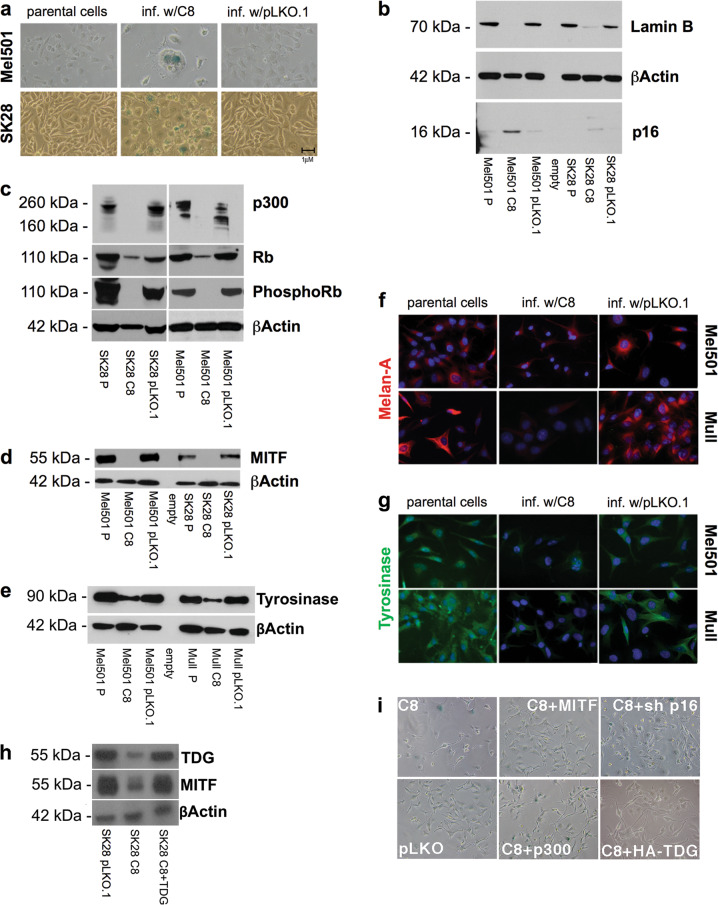
*TDG* knockdown induces senescence in melanoma. **a** Senescence-associated β-galactosidase activity assayed on day 3 postinfection in parental, C8-infected (*TDG* knockdown), and control pLKO.1-infected SK28 cells. SA-β-gal-positive cells are stained in blue. **b**–**d** Western blot showing the levels of lamin B, Rb, phospoRb, MITF, p300, and p16^INK4A^ in parental, C8-infected, and pLKO.1-infected Mel 501 and SK28 cells 3 days after infection. Lysates are from cells infected at the same time and conditions of those assayed by SA-β-gal staining. All the experiments were performed in duplicate and β-actin was used as a loading control. **e** Western blot detection of Tyrosinase in parental, *TDG* knockdown, and control pLKO.1-infected Mel501 and Mull cells. **f, g** Immunofluorescence detection of Melan-A/MART1 (upper panel, red) and Tyrosinase (lower panel, green) in parental, *TDG* knockdown, and control pLKO.1-infected Mel501 and Mull cell lines. Nuclei were counterstained with DAPI. Expression of shRNA-resistant TDG cDNA in C8-infected MEL501 cells rescues MITF levels (**h**) and morphological changes (**i**). Overexpression of MITF and p300 and p16^INK4^ knockdown suppress the morphological changes and proliferation arrest induced by *TDG* knockdown (**i**)

To determine whether the morphological changes (large, flat, dendritic process-bearing, multinucleated cells) observed upon *TDG* knockdown reflected senescence, we tested the expression of senescence-associated markers. Senescence-associated β-galactosidase assay (SA-β-gal) revealed positive staining in C8-infected Mel501 and SK28 cells (Fig. 3a). Consistent with this, western blot analysis showed an increase in the cyclin-dependent kinase inhibitor p16^INK4A^ and decreased lamin B1 (Fig. 3b), Rb, and phospho-Rb (Fig. 3c) in C8-infected Mel501 and SK28 melanoma cells, compared with parental and pLKO.1-infected cells. Decreased lamin B1 was confirmed by immunofluorescence (Fig. 2d).

TDG binds to and affects the activity of p300, a transcriptional coactivator that can acetylate each of the core histones as well as non-histone proteins such as p53, Rb, and E2F and is involved in cellular proliferation, differentiation, and apoptosis [2, 20, 21]. Reduction of p300 levels is known to induce senescence [22]. After *TDG* knockdown, p300 levels are low to undetectable (Fig. 3c) (As pLKO.1 vector-infected cells exhibit subtle changes in Rb and p300 expression in comparison to parental cells, it is possible that the lentiviral infection per se causes some changes; however, the difference in cellular and molecular phenotypes between pLKO.1- and C8-infected cells is very dramatic.).

To obtain further insight into the mechanism of senescence, we tested the expression of MITF, a master regulator of melanocyte development and a melanoma oncogene [23], whose silencing has been shown to induce senescence [24]. MITF expression was dramatically reduced in C8-infected Mel501 and SK28 melanoma cells (Fig. 3d). MITF is also the main transcriptional regulator of melanoma markers Tyrosinase and Melan-A/MART1 [23, 25]. We detected a dramatic abatement of Tyrosinase and Melan-A/MART1 expression upon *TDG* knockdown (Fig. 3e–g). Thus reduced MITF expression after *TDG* knockdown helps explain how these cells undergo senescence. Overexpression of an shRNA-resistant TDG cDNA rescued both MITF expression and morphological changes (Fig. 3h).

We next determined the role of p16^INK4A^, p300, and MITF in *TDG* knockdown-induced senescence. We found that overexpression of MITF and p300 in C8-infected SK28 cells rescues both the proliferation arrest and the morphological changes associated with *TDG* knockdown but not the formation of SA-β-gal-positive cells (Fig. 3i). We also found that co-infection of SK28 cells with shRNA lentiviruses against p16 ^INK4A^ and TDG (C8) rescued the proliferation defect. Thus the senescence induced by *TDG* knockdown was largely (with the exception of SA-β-gal-positivity) suppressed by p16^INK4A^ silencing and MITF and p300 expression (Fig. 3i).

### *TDG* knockdown affects genes involved in cell cycle and senescence

The above results show that *TDG* knockdown causes cell cycle arrest, senescence, and cell death. Given the role of TDG in transcription [2], to gain insight into the changes in gene expression associated with *TDG* knockdown, we compared the transcriptomes between control and C8-infected SK28 cells 5 days after infection by RNA-seq. Using a log2 fold change and adjusted *p* value, *TDG* knockdown had an important effect on gene expression with more than 1100 significantly downregulated and 1000 upregulated genes (see Supplemental Dataset).

**Fig. 4:**
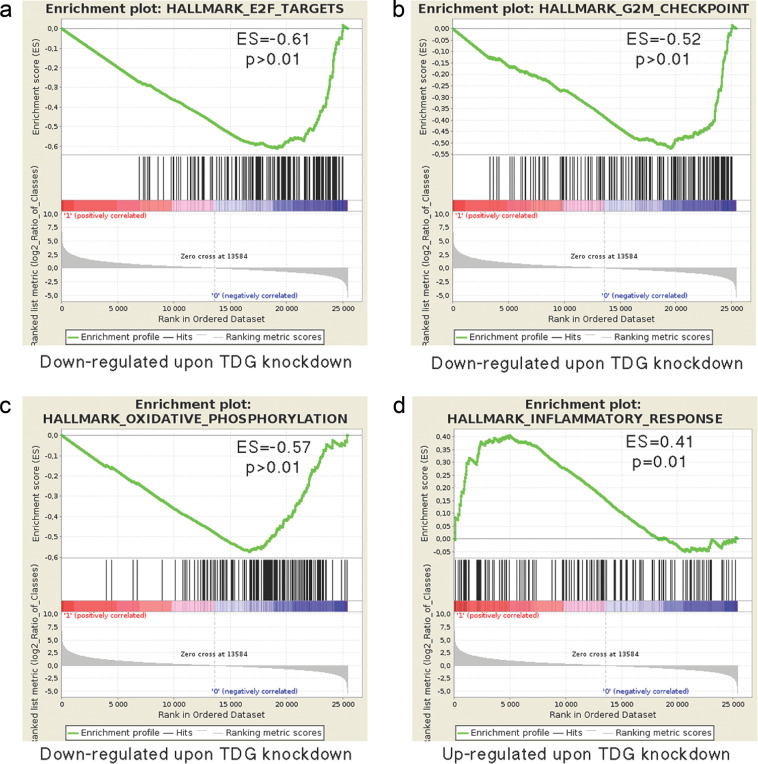
*TDG* knockdown affects genes involved in cell cycle regulation, oxidative phosphorylation, and senescence-associated secretory phenotype (SASP). **a**–**d** Gene set enrichment analysis (GSEA) of differentially expressed gene sets in C8-infected (*TDG* knockdown) and control pLKO.1-infected SK28 cells. The most significant categories in the upregulated and downregulated gene sets are shown

Gene set enrichment analysis (GSEA) revealed marked downregulation of E2F target genes involved in cell cycle progression and DNA replication and repair in C8-infected SK28 cells compared with control pLKO.1-infected cells (enrichment score (ES) = −0.61, familywise error rate *p* value >0.01; Fig. 4a). Similarly, genes associated with G2–M checkpoint are also diminished (Fig. 4b); the potent downregulation of these genes reflects the arrested cell cycle of *TDG* knockdown cells and their entry into senescence [26]. Knockdown of TDG also led to altered metabolic status with reduced expression of genes involved in oxidative phosphorylation (Fig. 4c); the critical role of oxidative phosphorylation in promoting melanoma growth has been recently highlighted by elucidation of the role of SAMMSON [27, 28].

GSEA also revealed upregulation of gene sets designated by the terms “TNFα signaling via NFκB” or “Inflammatory response” (ES, 0.41, *p* value 0.01; Fig. 4d); gene ontology (GO) analysis indicates that many of the genes upregulated in this group encode immune regulators and secreted cytokines and proteases that are known to be part of the senescence-associated secretory phenotype (SASP) [29–31]. The heatmaps that compare expression levels for individual genes in each of the four GSEA categories are shown in Suppl. Figure 5.

Thus *TDG* knockdown SK28 melanoma cells undergo senescence by repression of cell cycle progression genes and expression of SASP genes.

### *TDG* knockdown alters the epigenome by increasing DNA methylation

**Fig. 5:**
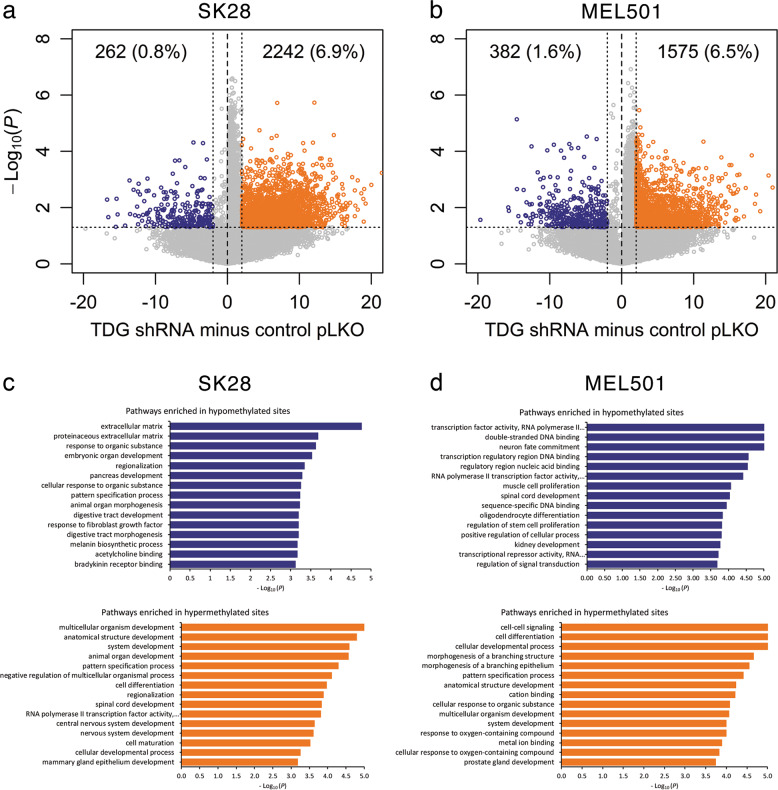
*TDG* knockdown affects the epigenome. Volcano plots show CpG sites differentially methylated by *TDG* knockdown in SK28 (**a**) and MEL501 (**b**) melanoma cells; blue and orange dots show hypomethylated and hypermethylated sites, respectively. The percentage of CpG sites undergoing changes in methylation upon *TDG* knockdown is indicated. Pathway analysis of genes mapping next to hypomethylated and hypermethylated CpG sites in SK28 (**c**) and MEL501 (**d**) cells. *x* axis: –log_10_(*p*)

Given the role of TDG in DNA demethylation, we began to investigate whether *TDG* knockdown alters the epigenome of melanoma cell lines. DNA methylation analysis (by digital restriction enzyme analysis of methylation (DREAM)) [32] of SK28 and MEL501 melanoma cells infected with TDG shRNA or control lentiviral vector revealed that, upon *TDG* knockdown, their methylomes were dramatically altered, as evidenced by the 6–7% increase in hypermethylated CpG sites and marginal increase in hypomethylated CpG sites (Fig. 5a, b). A GO analysis of genes mapping next to differentially methylated (hypo/hypermethylated) CpG sites in SK28 and MEL501 cells showed the involvement of pathways of transcriptional regulation, development, proliferation, and differentiation (Fig. 5c, d). Thus, in keeping with its role in DNA demethylation, *TDG* knockdown alters the epigenome by increasing DNA methylation.

### *TDG* knockdown reduces tumor growth in vivo

**Fig. 6:**
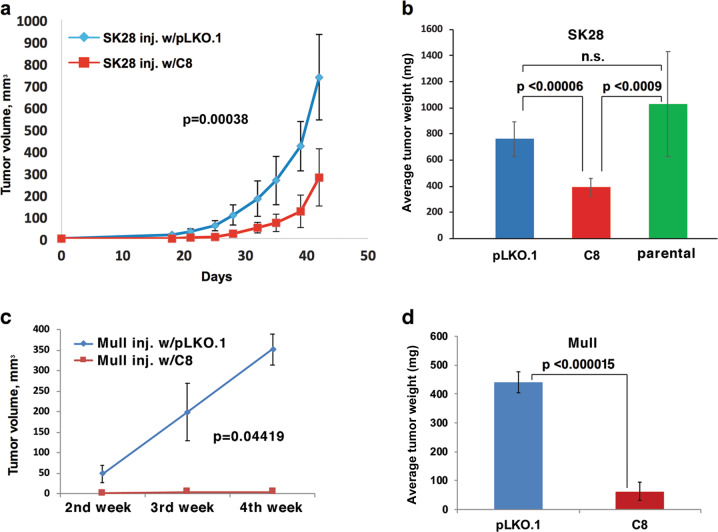
*TDG* knockdown inhibits the tumorigenic potential of SK28 and Mull melanoma cells. **a** Tumor growth curves of xenografts of control pLKO.1 vector-infected or C8-infected (*TDG* knockdown) SK28 cells injected subcutaneously into the flank of NSG mice. Data are presented as average ± standard deviation. **b** Average weight, at the end of the experiment, of tumors from xenografts of parental, control pLKO.1 vector-, and C8-infected SK28 cells. *p* Value was calculated by Fisher’s exact test; n.s. not significant. **c** Tumor growth curves of xenografts of control pLKO.1 vector-infected or C8-infected (*TDG* knockdown) Mull cells injected subcutaneously into the flank of NSG mice. **d** Average weight, at the end of the experiment, of tumors from xenografts of parental, control pLKO.1 vector-, and C8-infected Mull cells

To investigate whether *TDG* knockdown affected tumor formation in vivo, parental, C8-, and pLKO.1-infected SK28 cells were evaluated for tumor formation in NGS mice by injecting them subcutaneously into the flanks of six mice for each study arm. Tumors from *TDG* knockdown SK28 xenografts grew significantly smaller than tumors from control SK28 xenografts (Fig. 6a), and at the end of the study, the average tumor weight of *TDG* knockdown xenografts was approximately half of control pLKO.1 or parental xenografts (*p* < 0.00006 and *p* < 0.0009, respectively) (Fig. 6b). Similar results were obtained with the Mull cell line (*p* < 0.000015; Fig. 6c, d). Thus *TDG* knockdown reduces the ability of melanoma cells to form tumors in xenotransplant assay.

### Lack of toxicity of *TDG* inactivation in non-transformed cells/tissues

**Fig. 7:**
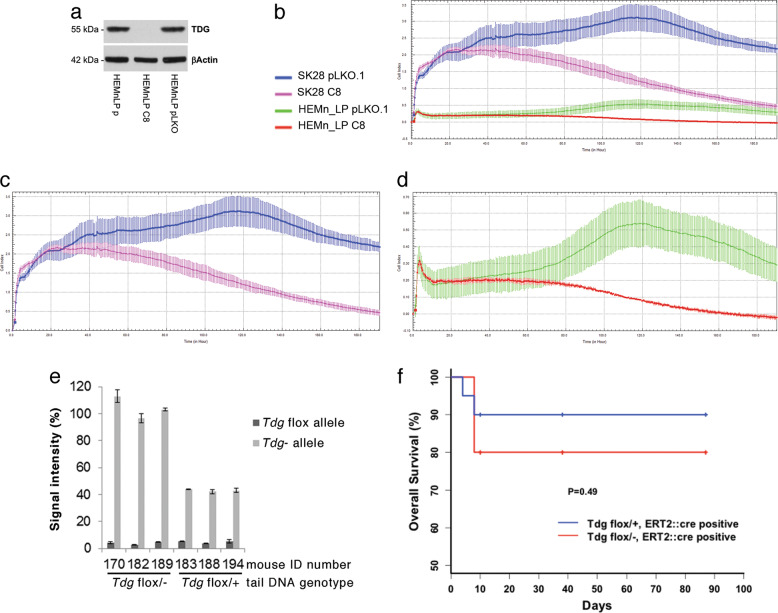
Lack of toxicity of *TDG* inactivation in non-transformed cells and adult mice. **a** Western blot showing effective *TDG* knockdown in C8-infected (*TDG* knockdown) but not in control pLKO.1-infected HEMn-LP normal, non-transformed melanocytes. **b** Cell proliferation analysis over an incubation time of 190 h by xCELLigence real-time cell analyzer in C8- and pLKO.1-infected HEMn-LP melanocytes and SK28 melanoma cells. All the experiments were performed in duplicate and are presented as average ± standard deviation. The same proliferation data are shown for SK28 melanoma cells only (**c**) and HEMn-LP melanocytes only (**d**). **e** Quantitation by qPCR of Cre-mediated recombination of the *Tdg*^flox^ allele and conversion into *Tdg*− allele in the blood from Cre-*ER*^*T2*^ *Tdg*^flox/−^ and Cre-*ER*^*T2*^ *Tdg*^flox/+^ mice. The data are expressed as percentage of signal intensity in comparison to that of the Anf gene. **f** Kaplan–Meier analysis of experimental *Tdg*^flox/−^-Cre-*ER*^*T2*^ mice (*n* = 30) and control *Tdg*^flox/+^-Cre-*ER*^*T2*^ mice (*n* = 15); time zero corresponds to tamoxifen administration. *p* Value is calculated by Fisher’s exact test

These results suggest that *TDG* inactivation may represent a viable anticancer strategy but raise the possibility of toxic effects on non-transformed cells. To test the effect of *TDG* inactivation in non-transformed cells in vitro, we knocked down *TDG* in HEMn-LP (Fig. 7a), a non-transformed melanocytic cell line, and compared their proliferation to that of *TDG* knockdown SK28 cells, over an incubation time of 190 h by xCELLigence real-time cell analyzer. Starting at 55 h, in comparison to matched pLKO-infected cells, *TDG* knockdown significantly decreased the proliferation of SK28 melanoma cells but had a smaller effect on normal melanocytic cells, starting at ~ 80 h, perhaps due to their lower growth rate (Fig. 7b–d; Suppl. Figure 6).

To investigate the effects of *TDG* inactivation in normal adult tissues and rule out potential toxic effects in vivo, we generated conditional *Tdg* knockout mice, bypassing embryonic lethality associated with germline *Tdg* inactivation [2, 4]. We used Cre-*ER*^*T2*^ transgenic mice in which tamoxifen-inducible Cre recombinase is driven by the ubiquitous human *UBC* promoter [33]; thus inactivation of the *Tdg*^flox^ allele would be expected in most tissues of the mice. A total of 45 mice were divided into two genotype groups: experimental Cre-*ER*^*T2*^ *Tdg*^flox/−^ and control Cre-*ER*^*T2*^ *Tdg*^flox/+^. After tamoxifen administration by oral gavage, the deletion of *Tdg*^flox^ allele was confirmed by quantitative PCR (qPCR) on DNA extracted from blood (Fig. 7e). Cre-*ER*^*T2*^ *Tdg*^flox/−^ and Cre-*ER*^*T2*^ *Tdg*^flox/+^ mice were aged and monitored for neoplastic lesions or general morbidity over a 3-month period after tamoxifen administration. Early deaths occurred within the first 10 days (Fig. 7f) and could be attributed to non-specific consequence of the tamoxifen gavage; in fact, oral gavage is known to be associated with a 15% mortality, due to esophageal perforation or gastro-esophageal reflux and consequent pneumonia [34, 35]. Upon further aging, no significant differences in survival were observed between the two genotype groups (Fig. 7f). At 3 months after tamoxifen treatment, mice were sacrificed and inspected for the presence of tissue abnormalities or gross phenotypes. No significant macroscopic differences were found between experimental Cre-*ER*^*T2*^ *Tdg*^flox/−^ mice and control Cre-*ER*^*T2*^ *Tdg*^flox/+^ mice. However, in 2/4 Cre-*ER*^*T2*^ *Tdg*^flox/−^ mice for which histopathological analysis was conducted, a histiocytic proliferation was detected in the liver and spleen; histiocytic proliferation was not found in 0/4 Cre-*ER*^*T2*^ *Tdg*^flox/+^ mice that underwent histopathological analysis (Suppl. Figure 7). The significance of these histopathological findings and their relationship to TDG inactivation are unclear, because histiocytosis has been reported to be associated with oral gavage [35].

Thus in vitro and in vivo results indicate that, unlike tumor cells, TDG is dispensable in normal cells and tissues, which rules out significant toxicity in normal cells and tissues due to *TDG* inactivation.

### Identification of candidate TDG inhibitors

The antiproliferative effects of *TDG* knockdown in cancer cells raise the possibility that small molecule inhibitors that catalytically inactivate TDG may be useful for melanoma treatment. To identify inhibitors of TDG glycosylase activity, we employed an in vitro DNA repair molecular beacon assay in which a hairpin-shaped G:U-mismatched oligonucleotide is incubated with highly active preparations of recombinant TDG and recombinant AP endonuclease (APE1), the latter acting as a downstream coupling enzyme. In the folded hairpin substrate, the fluorescence of 5’-conjugated 6-FAM is quenched by a dabsyl “black hole” at the 3’ end [18]. Upon removal of the mismatched U by TDG, and incision of the resulting abasic/AP site by APE1, a short oligonucleotide containing 6-FAM is released away from the quencher. The resulting fluorescence is monitored on a real-time qPCR machine over a 2 h incubation at 37 °C, providing a sensitive and quantitative measurement of repair activity (Suppl. Figure 7a). Because the assay is bi-enzymatic, we first established the dose dependence of both APE1 and TDG in 384-well format (Suppl. Figure 7b-c).

**Fig. 8:**
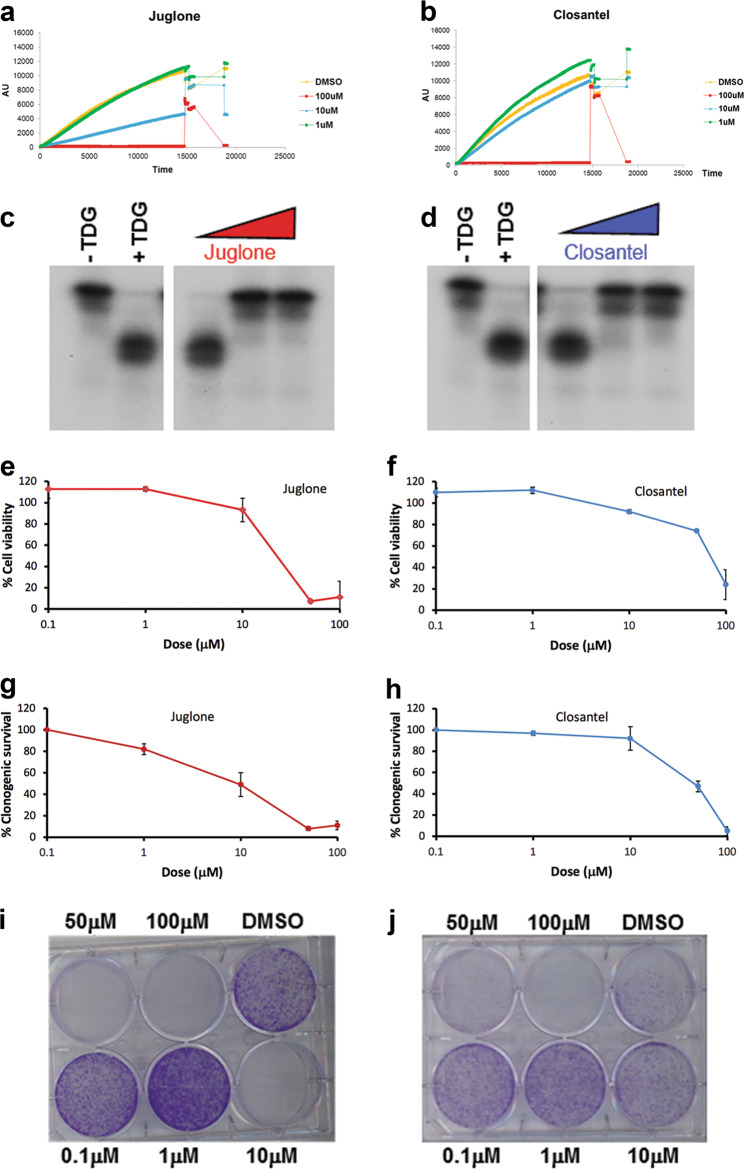

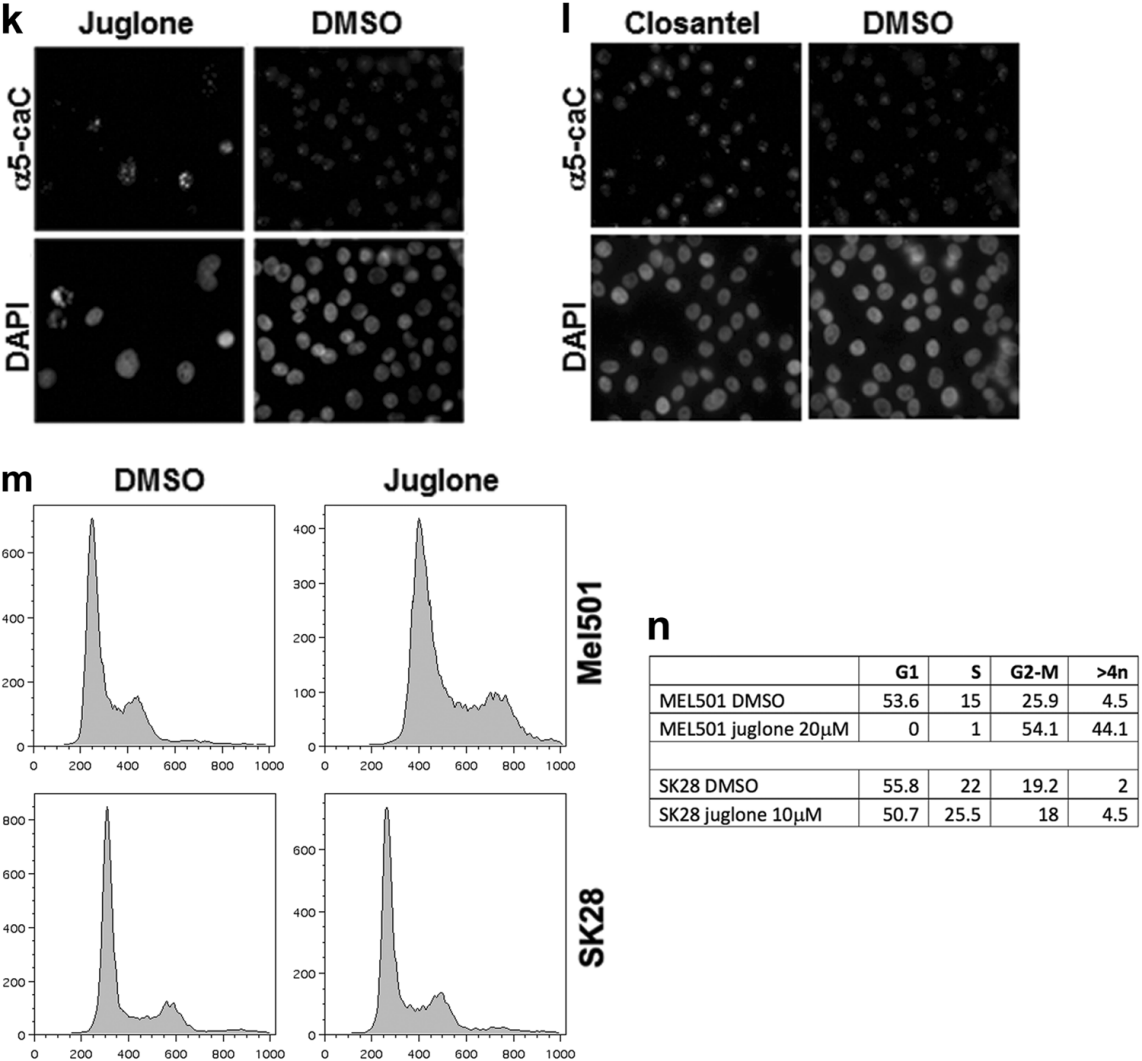
TDG inhibitors decrease clonogenic capacity and cell viability in melanoma cells. **a**, **b** Molecular beacon DNA repair assay showing dose-dependent inhibition of TDG glycosylase activity by juglone and closantel, respectively; fluorescence is in arbitrary units (AU); time is in 0.5-s intervals. **c**, **d** Conventional TDG glycosylase assays, in which G:T mismatch-bearing double-stranded oligonucleotide substrates and ^32^P-labeled at the 3′ end on the mismatched T-containing strand were treated with purified recombinant TDG protein at 37 °C; the reactions were then treated with 100 mM NaOH at 90 °C for 30’ in order to cleave the sugar-phosphate backbone at the AP site and the resulting reaction product was separated from the longer substrate by PAGE. Juglone and closantel were tested at concentrations of 100 nM, 10 μM, and 1 mM. A negative control is included. **e**, **f** Cell viability of SK28 melanoma cells treated with increasing doses of juglone and closantel. **g**, **h** Clonogenic capacity of SK28 melanoma cells treated with different doses of juglone and closantel or DMSO as a control; **i**, **j** corresponding stained plates. All the experiments were performed in duplicate or triplicate and data, normalized to DMSO, are presented as average ± standard deviation. **k**, **l** Detection by immunofluorescence of 5caC in juglone- and closantel-treated SK28 cells; DMSO was used as a negative control; nuclei were counter-stained with DAPI. **m**, **n**Fluorescence-activated cell sorting (FACS) of propidium iodide-stained Mel501 and SK28 cells, showing that juglone induces cell cycle arrest in G2–M phase and increased S phase/>4*n* cells, respectively

After optimizing the assay for 384-well format (*Z*’ score 0.6) [36], we screened the ICCB bioactive library (~500 compounds) and the Johns Hopkins Clinical Compound library (~1500 drugs), identifying 18 and 14 candidate inhibitors, respectively (Suppl. Tables 1 and 2; Fig. 8a, b). Subsequently, the TDG assay was successfully miniaturized in the 1536-well format (*Z*’ score 0.6) [36] and a new screen was conducted against the Sigma Aldrich LOPAC^1280^ compound library in a dose–response fashion that allowed us to compare the IC_50_ of compounds, identifying 16 positive hits, which upon elimination of promiscuous hits and DNA binders were reduced to 9 compounds (Suppl. Table 3). One of these compounds is aurothioglucose, a glucose derivative used for rheumatoid arthritis that had also been identified in the 384-well format screening. Some of these compounds, including closantel, juglone, and cefixime, were confirmed as TDG inhibitors in a standard, radioactive DNA glycosylase assay (Fig. 8c, d).

### Candidate TDG inhibitors reduce cell viability and clonogenic capacity in melanoma cells

We next performed MTS [3-(4,5-dimethylthiazol-2-yl)-5-(3-carboxymethoxyphenyl)-2-(4- sulfophenyl)-2H-tetrazolium] and clonogenic assays in juglone- and closantel-treated SK28 cells. After 24 h exposure to each drug, we detected a dose-dependent reduction of cell viability by MTS assays, with IC_50_ of approximately 10 μM for juglone and closantel (Fig. 8e, f). In agreement with the MTS results, we observed a dose-dependent reduction of colony formation in clonogenic assays, with IC_50_ of approximately 10 and 40 μM for juglone and closantel, respectively (Fig. 8g–j). To determine whether these two drugs target TDG at concentrations comparable to their IC_50_, nuclei of juglone- and closantel-treated SK28cells at 10 µM were stained for 5caC: the results showed increased 5caC staining compared to dimethyl sulfoxide (DMSO)-treated cells, confirming TDG inhibition (Fig. 8k, l). In addition, juglone treatment recapitulated the G2–M cell cycle arrest of *TDG* knockdown in MEL501 and increased the percentage of S phase and >4*n* cells in SK28 (Fig. 8m, n). Treatment of normal melanocytic cell line HEMn-LP with juglone and closantel elicited a reduction in viability (Suppl. Fig. 9). Together, these results demonstrate that TDG inhibition can arrest tumor cell growth and eventually induce cell death.

## Discussion

Our studies show that TDG is required for melanoma cell proliferation, survival, and tumor formation. *TDG* knockdown caused a robust cell cycle arrest, cell death, and a senescent phenotype. Gene expression profiling of *TDG* knockdown cells showed upregulation of known melanoma SASP genes, hallmark of senescence, and downregulation of cell cycle factors, such as E2F target genes and DNA replication genes, which may explain the cell cycle arrest.

*TDG* knockdown seems to affect melanoma cells irrespective of *TP53/BRAF/NRAS* mutational status; in fact MEL501, Mull, and SK28 harbor a *BRAF*-V600E mutation, whereas Gerlach cells are *NRAS* mutant, and MNT-1 are wild type for both *BRAF* and *NRAS*. However, the mutational background of a given cell line may determine different responses to *TDG* knockdown; for instance, the S-phase arrest of SK28 cells, as opposed to the G2–M arrest of MEL501 and Mull cells, may be related to the fact that SK28 cells contain a *TP53* mutation (L145R).

In addition to its role in DNA repair and active DNA demethylation, TDG has also been implicated in transcriptional regulation as co-activator of several transcription factors, including histone acetyltransferases p300 and CBP [2]. We recently reported that TDG promotes histone acetylation by p300 and affects p300 selectivity on specific histone H3 lysines (K18 and K23) in melanoma [20]. Interestingly, p300 and CBP acetylate MITF and act as MITF co-activators. Importantly, depletion of p300 and MITF in melanoma cells has been show to induce cell cycle arrest and a senescent phenotype [37–39]. A complex transcriptional network might also regulate the levels of these factors. In fact, MITF binds to the *TDG* promoter [29]. Thus it is possible that *TDG* knockdown causes senescence in melanoma cells by decreasing the levels and activity of p300 and MITF, which in turn may further deplete *TDG* by reducing its transcription. Overexpression of p300 and MITF rescues the proliferation arrest and morphological changes induced by *TDG* knockdown but not the SA-β-gal-positivity, thus confirming the decreased levels of p300 and MITF as a possible mechanism of *TDG* knockdown-induced senescence. Multinucleation, a feature of senescent cells expressing high levels of p16^INK4A^ (ref. [40]), was also seen as an adaptation to p300 silencing [41]. It is possible that the very low levels of Rb and lack of pRb in *TDG* knockdown cells may further enhance multinucleation. The rescue of the proliferation arrest by p16^INK4A^ knockdown indicates that the p16^INK4A^–Rb pathway is also involved in *TDG* knockdown-induced senescence, an issue that merits additional studies in future and that may clarify the relationship among proliferation arrest and multinucleation. The persistence of SA-β-gal-positivity after suppression of *TDG* knockdown-induced senescence by p16^INK4A^ silencing and MITF and p300 expression is interesting. It should be noted that SA-β-gal can be positive in biological conditions other than senescence (reviewed in ref. [42]); alternatively, as hypothesized for phosphoinositide-3 kinase/phosphatase and tensin homolog signaling [42], fine-tuning of TDG expression levels may be necessary to maintain cell proliferation and counteract senescence; therefore, in rescue experiments by transient transfection, cells that express just the appropriate amount of TDG for the desired rescue phenotype may be few.

The appearance of dendritic processes in melanoma cells upon *TDG* knockdown is a distinctive morphological feature that renders them similar to dendritic melanocytes, which are found in Braf^V600E^-induced nevi and stain positive for SA-β-Gal activity [43]. Also, it has been noted that senescent cells may adopt a dendritic shape [44]. Thus dendrites may be a morphological feature of *TDG* knockdown-induced senescence of melanoma cells.

Time-lapse microscopy of Mel501 cells showed that *TDG* knockdown also caused cell death following what is presumably a cell cycle arrest in G2 (as determined by FACS). The precise involvement of TDG in preventing these alterations not only requires additional studies but may also be related to the reduced levels of p300, as depletion of p300, CBP, or P/CAF leads to mitotic catastrophe in human cell lines [41].

We found that *TDG* knockdown alters the epigenome by increasing hypermethylated CpG sites by 6–7%, in keeping with its role in DNA demethylation. It was thought originally that, when cells were treated with the DNA demethylating agents azacitidine or decitabine, genome-wide demethylation would cause significant toxicity. However, it was later found that only select regions of the genome were demethylated by these drugs [45, 46]. Here the same thing, but in a specular way, occurs after TDG inactivation: a small but significant fraction of the genome undergoes hypermethylation, which suggests that TDG inhibitors will not cause non-specific and generalized toxicity through massive genome hypermethylation.

Importantly, *TDG* knockdown strongly suppresses the tumor-forming capabilities of melanoma lines in xenograft assays, suggesting that one or more of TDG activities are critical for tumor induction, maintenance, and/or progression and therefore may potentially represent a novel vulnerability of melanoma.

The miniaturized screening assay that we devised, amenable to high-throughput applications, led to the identification of first-generation TDG inhibitors that reduced viability and clonogenic capacity of melanoma lines. Since these inhibitors have IC_50_ in the low micromolar range and have hit in other screening campaigns, we cannot rule out off-target effects. However, they must inhibit TDG in cellular context, because they increase the normally undetectable levels of 5caC. First-generation TDG inhibitors also reduced viability of normal melanocytes, which may also be a consequence of off-target effects.

While some of these inhibitors are Food and Drug Administration approved, such as aurothioglucose, others are not suitable for human use (juglone, closantel). Our findings nonetheless highlight the utility of our screen in identifying chemicals that can inhibit TDG activity. It is not clear whether the drugs identified could be repurposed or modified by medicinal chemistry, as opposed to looking for more potent and specific hits by screening larger compound libraries. Identification of more potent and specific inhibitors of TDG is probably a better strategy to overcome the cytotoxicity on normal melanocytes (Suppl. Fig. 9). However, the fact that untransformed melanocytes are minimally affected by *TDG* knockdown and only show reduced proliferation starting at 80 h (whereas melanoma cells have marked reduction in cell index starting at 55 h, Fig. 7b–d), and that conditional knockout of *TDG* in adult mice is well tolerated (Fig. 7f), rules out significant toxicity in normal cells/tissues due to specific *TDG* inactivation and indicates that it should be possible to achieve an effective therapeutic window. In fact, evidence so far accumulated suggests that the normal role of TDG may be limited largely to development and that therapeutic targeting may spare adult somatic cells.

Future experiments will address in more detail the specific requirement of TDG for cancer cells, which, based on particular mutations and epigenetic alterations of the individual tumor, should both enable the identification of patients more likely to benefit from a therapy with TDG inhibitors and begin to establish rational basis for combinatorial treatments of TDG inhibitors with existing antimelanoma drugs.

## Materials and methods

### Cell culture

SK28, 888-Mel (from ATCC), Mull, Rosi (from Benoit van den Eynde), Mel501 (from Colin Goding), and MNT-1 (from Graça Raposo) were validated by comparative genomic hybridization and cultured in RPMI-1640 supplemented with 10% fetal bovine serum, 1% penicillin/streptomycin, and 1% L-glutamine. A non-transformed epidermal melanocytic line HEMn-LP (from Raza Zaidi) was cultured in Medium 254 (Invitrogen) supplemented with Human Melanocyte Growth Supplement (Invitrogen).

### Western blot analysis

Western blotting was conducted as previously described [20]. Membranes were incubated with antibodies anti-TDG, anti-lamin B1 (both Abcam), anti-p16^INK4A^ (Santa Cruz Biotechnology), anti-p300 (Calbiochem), anti-Rb, anti-phosphoRb (both Cell Signaling), anti-MITF (Interchim), and anti-β-actin (Sigma).

### ***TDG*** knockdown by lentiviral infection

Two different *TDG* shRNA lentiviral constructs, named C8 (Thermo Scientific Open Bio-Systems) and sh4575 (Sigma-Aldrich), and the empty pLKO.1 lentiviral vector were used in this study. *TDG* knockdown was performed as previously described [2]. Control pLKO.1 and *TDG* shRNA lentivirus-infected melanoma cell lines were selected in puromycin (0.5 μg/mL) for 3–5 days. For the experiments of overexpression of TDG, MITF, and p300, infections were conducted 24 h after transfection of these clones (by Lipofectamine 2000, Thermo Fisher); 4–5 days later, the transfected/infected cells were fixed for SA-β-gal activity (see below). Transfection/infection experiments were repeated four times.

### Cell cycle analysis

Cell cycle analysis was conducted as previously described [47] by collecting both attached cells and cells floating in the medium. Samples were analyzed in biological triplicate with a FACScan (Becton Dickinson) using the FLOW-JO software (Tree Star, Ashland, OR). Cell cycle quantitation was conducted with the Dean/Jett/Fox method, using FLOW-JO.

### Cell viability assay

SK28 cells, seeded in 96-well plates (at 2000–5000 cells/well), were treated with vehicle or drugs at the indicated concentrations for 24, 48, or 72 h. Viability was measured with MTS, using the Cell Titer 96 Assay (Promega). Absorbance was measured in a Synergy HT plate reader (Biotek). All experiments were conducted in triplicate.

### Clonogenic assay

Clonogenic capacity was determined as previously described [47]. Briefly, 24 h after seeding, DMSO (vehicle control) or drugs at the indicated concentrations were added for 24 h. Drugs were removed and replaced with drug-free medium. After 10–14 days, the cells were stained with Clonogenic Dye (4% Crystal Violet powder in 20% EtOH) for 30’. Individual colonies were counted. Each clonogenic assay was tested in triplicate or quadruplicate.

### Cell proliferation

Cell proliferation was measured with the xCELLigence Real-Time Cell Analysis, using modified 16-well E-plates (ACEA Biosciences, Inc., San Diego, CA). SK28 and HEMn-LP cells were seeded at 4 × 10^3^ and 1.5 × 10^4^ cells/well, respectively, and proliferation was monitored in real-time over 81 h. Each condition was tested in duplicate.

### β-Galactosidase activity

SA-β-gal activity was detected at pH 6.0 [48]. Mel501 and SK28 cells, plated into a 6-well plate onto cover slips at 1–1.5 × 10^5^ cells/well, were stained with 40 mM Na_2_HPO_4_, pH 6.0, 150 mM NaCl, 2 mM MgCl_2_, 5 mM K_3_Fe(CN)_6_, 5 mM K_4_Fe(CN)_6_, and 1 mg/mL 5-bromo-4-chloro-3-indolyl β-D-galactopyranoside (X-gal) at 37 °C for 5–7 h in the dark into a non-CO_2_ incubator. Cells were fixed for 5’ at room temperature in 2% formaldehyde/0.2% glutaraldehyde in phosphate-buffered saline (PBS), rinsed in PBS and eventually in ddH_2_O, and then photographed with a phase-contrast microscope.

### Immunofluorescence

Cells were seeded onto acid-washed coverslips and cultured for at least 24 h before fixation and staining. Cells were fixed (4% paraformaldehyde in PBS for 10’), permeabilized for 1–2’ at room temperature with 0.2% Triton X-100 in KB buffer (20 mM Tris-HCl, pH 7.5, 150 mM NaCl, and 1% bovine serum albumin (BSA)), washed in KB, and blocked with 3% BSA in PBS for 1 h. Primary antibodies anti-5caC (Active Motif), anti-tyrosinase, and anti-Melan-A (both from Santa Cruz Biotechnology) were incubated overnight at 4 °C. After washing twice in KB, samples were incubated with Alexa Fluor-conjugated anti-rabbit (Molecular Probes) for 30’. Nuclei were counterstained with DAPI. The nuclear envelope was detected by immunofluorescence staining of lamin B using a mouse monoclonal antibody (Abcam) and following protocol provided by the vendor. Images were taken using a Leica Microsystems TCS-SP8A confocal microscope controlled by the LAS software. Exposure times were optimized for control samples and identical exposure times were used for experimental samples.

### Time-lapse videomicroscopy

Forty-eight hours after lentiviral infection, Mel501 cells stably expressing GFP:histone H2B were seeded into 6-well plates in complete RPMI medium+puromycin, supplemented with HEPES (25 mM), layered with mineral oil (Sigma), and placed into housing chamber at 37 °C. Images were acquired every 15’ for 72 h on Nikon TE300 inverted epi-fluorescent microscope (Nikon Instruments) with Retiga EXi CCD camera (QImaging). Images were compiled into video using the MetaMorph software (Molecular Devices). Cell fates were analyzed frame by frame.

### Xenografts

SK28 cells were resuspended within Matrigel and injected in SCID/NSG female mice (5 × 10^6^ cells/flank). Mice were observed daily and tumor volumes were measured twice a week with digital caliper. Tumor volumes were calculated as: (length × width^2^)/2 (ref. [49]). When xenografts in vehicle-treated mice reached ~1.5 cm diameter, mice were sacrificed. We referred to similar published studies in which six animals per arm are used. Mice were housed in the Fox Chase Cancer Center Laboratory Animal Facility, a fully accredited facility, and all experiments were approved by the Fox Chase Cancer Center Institutional Animal Care and Use Committee.

### Conditional inactivation of *Tdg* in adult mice

For conditional inactivation of *Tdg* in adult mice, we crossed Cre-*ER*^*T2*^ transgenic mice [33] (gift of Eric Brown) with *Tdg*^flox/flox^ conditional knockout mice [50]. All the strains used have a nearly pure C57BL/6 genetic background (at least 10 backcrosses in this background). To inactivate the *Tdg*^flox^ allele, we treated Cre-*ER*^*T2*^ *Tdg*^−/flox^ mice and control Cre-*ER*^*T2*^ *Tdg*^flox/+^ mice with tamoxifen (Sigma T-5648), 20 mg/mL in corn oil (Sigma C-8267), by oral gavage, three times per mouse every other day, using a 1 mL syringe and a 20-G sterile 1.5” curved feeding needle with 2.25 mm ball (Braintree Scientific N-PK 01022). Total administration of tamoxifen was 6 mg per mouse. Tamoxifen treatments were conducted on randomized animals (genotype unknown to the experimenter).

After 3 weeks from the last gavage, *Tdg* genotyping was performed on DNA extracted from blood collected from retro-orbital bleeding by the QIAamp DNA Blood Mini Kit (Qiagen). Experimental Cre-*ER*^*T2*^ *Tdg*^flox/−^ and control Cre-*ER*^*T2*^ *Tdg*^flox/+^ mice with an extensive *Tdg*^flox^ allele deletion, as determined by qPCR, were included in this study, monitored weekly, and sacrificed at the first development of signs of distress or at a maximum of 90 days. A Kaplan–Meier analysis was performed and statistical significance was measured using log-rank test.

### RNA sequencing

RNA (biological triplicates) was prepared with the RNeasy Mini Kit (Qiagen), and quality control was performed on a Bioanalyzer. Messenger-RNA-seq was performed essentially as described [51]. Sequence reads were mapped to reference genome mm9/NCBI37 using Tophat v2.0.10 [52] and the bowtie2 v2.1.0 aligner [53]. Only uniquely aligned reads were retained for further analyses. Data normalization and quantification of gene expression was performed using the DESeq2 Bioconductor package. Significantly deregulated genes were selected using a log2 fold change >1 and <1 and adjusted *p* value cutoff of 0.05. GO analyses were performed using DAVID functional annotation clustering tool (https://david.ncifcrf.gov/summary.jsp). For GSEA, we used the mean of the log2 fold changes of the biological replicates as metric for the H Hallmark gene sets of the BROAD javaGSEA tool with 1000 permutations and the canonical pathway subcollection of the C2-curated BROAD molecular signature gene set collection. Comparisons of interest were performed [54] as implemented in the DESeq2 Bioconductor library (v1.0.18). Resulting *p* values were adjusted for multiple testing by the Benjamini and Hochberg method [55]. Heatmaps were made using the log2 of RPKM values with R package “pheatmap” v1.0.10 using options scale = ‘none’ and cluster_rows = TRUE. For heatmaps of deregulated genes, only the 300 most upregulated or downregulated genes were represented.

### Analysis of DNA methylation by DREAM

DREAM was performed to determine the methylation profile of MEL501 and SK28 melanoma cell lines (biological duplicates). Briefly, genomic DNA spiked with methylation standards was sequentially digested by SmaI and XmaI, which both recognize the sequence CCCGGG. SmaI is methylation sensitive, whereas XmaI is methylation insensitive. Distinct signatures, 5’-GGG at unmethylated sites or 5’-CCGGG at methylated sites, were created by enzyme digestion. 3’ recesses were filled in, and Illumina adaptors were ligated to the ends of restriction fragments. Barcoded library pools were sequenced using Illumina HiSeq2500 instrument. Illumina fastq files were aligned to the human genome (hg19) using Bowtie2 [53]. Uniquely mapped reads were sorted into bam files using SAMtools [56, 57]. We used a Python script to map aligned sequencing reads to target CCCGGG sites in the human genome and enumerate signatures corresponding to methylated (starting with CCGGG) and unmethylated (starting with GGG) CpG for each target site. We calculated methylation levels at each SmaI/XmaI site based on the percentage of methylated signatures. Background-corrected GO analyses were performed using ConsensusPathDB (http://cpdb.molgen.mpg.de).

### Screening assay

TDG/APE1 activity was measured using a G:U-mismatched DNA repair molecular beacon in which the substrate adopts a hairpin structure, with quenching upon folding and fluorescence release upon base removal/cleavage at the lesion site [18]. The substrate (Biosearch Technologies, Novato, CA) was: 5’dT(FAM)-CCACT-d-Uridine-GTGAATTGACAGCCCATGTGCATCAATTCACGAGTGG-T(Dabsyl)3’, where FAM and Dabsyl are conjugated to the thymine C5 carbon, to avoid degradation of the fluorophores by the exonuclease of APE1 [58]. The substrate was allowed to fold by heating at 94 °C for 3’ and cooling to 4 °C at 0.1 °C/s at 100 µM in 4× BER buffer (1×: Hepes-KOH 25 mM, KCl 150 mM, Glycerol 1%, DTT 0.5 mM). Recombinant APE1 (NEB) and recombinant TDG [2] were diluted to 0.5 and 0.25 µM respectively in 40 mM Hepes-KOH, 100 mM NaCl, and 10% glycerol. Substrate and enzymes were mixed and dispensed in 384-well plates (10 µL/well) to final concentrations of 280 nM (substrate), 30 nM (TDG), and 60 nM (APE1) in 1× BER buffer supplemented with MgCl_2_ (1.2 mM), DTT (10 mM), and BSA (0.5 mg/mL, NEB). The ICCB Known Bioactives Library (Enzo Life Sciences) and the Johns Hopkins Clinical Compound Library [59] were screened. Compounds varied in concentration from 0.1 mM to 10 mM. Compounds dissolved in DMSO were added to wells of 384-well plates by pin-tool transfer on a CyBi-Well Vario (Analytik Jena AG, Jena, Germany) equipped with a 25 nL pin-tool (V and P Scientific). The reaction was initiated by bringing the plate to 37 °C in a real-time PCR machine (Applied Biosystems 7900 HT). FAM fluorescence was recorded for 2 h at 0.5-s intervals.

### Assay miniaturization in 1536-well format

To utilize fully automated robotic platforms for dose–response screening, the previous assay was miniaturized into 1536-well format by not only miniaturizing the reaction volume (4 µL) but also by optimizing enzyme concentrations. Briefly, 3 µL of reagents (buffer as negative control and 100 nM of TDG and 3 nM of APE1) were dispensed by Flying Reagent Dispenser™ (Beckman Coulter Inc.) into 1536-well plates. Compounds were delivered as 23 nL in DMSO via pin-tool transfer; vehicle-only control consisted of 23 nL DMSO. The plate was incubated for 15’ at room temperature, and then 1 µL of folded hairpin substrate (100 nM) was added to start the reaction. The plate was immediately transferred into ViewLux reader to measure fluorescence signal at 0 and 3 h. IC_50_ values were calculated from dose–response curve fits using the fluorescence intensity change, relating it to uninhibited and no-enzyme controls, respectively. The assay was used to screen the LOPAC^1280^ library of bioactive compounds (Sigma-Aldrich).

### DNA glycosylase assay

DNA glycosylase assays were conducted as previously described [2, 60] on a G:U mismatched substrate obtained by annealing the oligonucleotides CAATCCTAGCTGACACGATGTGGCCAATGGCATGACT and GAGTCATGCCATTGGCCACATUGTGTCAGCTAGGATT [61], in which the latter was radiolabeled at the 5′ end with T4 polynucleotide kinase (New England Biolabs) and γ-^32^P-ATP (NEN Dupont). The reactions, containing vehicle or candidate inhibitors at increasing concentrations, were incubated at 37 °C for 30’ and then treated with NaOH at 90 °C for 30’ to cleave the abasic site. Substrate and product bands were separated by 8.3 M urea/25% polyacrylamide gel electrophoresis and exposed to autoradiography.

### Bioinformatics analysis

Oncomine 4.5 database (http://www.oncomine.org) was used to investigate the expression of *TDG* in normal skin, nevi, and cutaneous melanoma samples in the Talantov melanoma dataset. The Human Protein Atlas pathology database (http://www.proteinatlas.org) was used to assess TDG protein expression in human melanoma and to investigate the correlation between *TDG* mRNA expression level and patient survival by Kaplan–Meier analysis.

## Supplementary information

Supplementary Figure Legends_clean

Supplementary Figure 1

Supplementary Figure 2

Supplementary Figure 3

Supplementary Figure 4

Supplementary Figure 5

Supplementary Figure 6

Supplementary Figure 7

Supplementary Figure 8

Supplementary Figure 9

Supplementary Tables 1-2-3

Supplementary Dataset RNA seq

## Data Availability

The datasets generated during and/or analysed during the current study have been submitted to the GEO repository at accession numbers GSE123681 and GSE123928.
