# Supplementary material for: Thymine DNA glycosylase as a novel target for melanoma
Source: Oncogene. 2019 Jan 23;38(19):3710–28. doi: 10.1038/s41388-018-0640-2 (PMC6563616; doi:10.1038/s41388-018-0640-2)
Supplement: Supplementary file 1 — Supplementary Figure Legends_clean [file 41388_2018_640_MOESM1_ESM.doc]

**SUPPLEMENTAL FIGURE LEGENDS**

**Supplementary Figure 1**

(a) Kaplan-Meier plots showing the correlation between TDG mRNA expression level and patient survival. Patients were divided based on TDG expression level into two groups indicated as "low" or "high". Log-rank P value and 3-year survival for patients with high and low *TDG* expression are shown (from Protein Atlas Database). (b) Correlation between *TDG* and *TET1-3* mRNA expression levels in TCGA-SKCM.

**Supplementary Figure 2**

(a) Western blotting showing TDG expression in a panel of tumorigenic (blue) and non-tumorigenic (red) human melanoma cell lines. (b) Western blotting showing that TDG is expressed in normal human melanocytes HEMn-LP and HEMn-MP at levels similar to human melanoma cell lines. (c) TDG mRNA expression (normalized probe intensity) in a panel of tumorigenic (blue) and non-tumorigenic (red) human melanoma cell lines; expression in normal human melanocytes (white) is also shown.

**Supplementary Figure 3**

Percentage of cells showing one or more nuclei in parental, C8-infected (*TDG* knockdown) and control pLKO.1-infected Mel501 and Mull cells.

**Supplementary Figure 4**

(a) Phase-contrast images of parental, *TDG* knockdown (C8) and control pLKO.1-infected Rosi and MNT-1 melanoma cells. (b) Western blot showing effective *TDG* knockdown in sh4575 lentivirus-infected Mel501 cells in comparison to parental and control pLKO.1-infected cells, 3 days after infection. (c) Phase-contrast images of parental and sh4575 lentivirus-infected Mel501 cells. (d) Immunofluorescence staining for 5-carboxylcytosine (5caC) in sh4575 lentivirus-infected (*TDG* knockdown) and control pLKO.1-infected Mel501 cells. Nuclei are counter-stained with DAPI.

**Supplementary Figure 5**

Heatmaps comparing expression levels for individual genes in each of the four indicated GSEA categories.

**Supplementary Figure 6**

Cell proliferation analysis over an incubation time of 170 hours by xCELLigence real-time cell analyzer in C8- (green) and control pLKO.1-infected (red) SK28 cells. All the experiments were performed in duplicate and data are presented as average ± standard deviation.

**Supplementary Figure 7**

Representative hematoxylin & eosin-stained histopathology sections from the liver and spleen of two mice with the Cre-*ER^T2^ Tdg*^flox/-^genotype and one mouse with the control Cre-*ER^T2^ Tdg*^flox/+^ genotype, as indicated. Images were photographed with a 20x objective. Scale bar is shown. Arrows mark histiocytic proliferation in the portal spaces of the liver and within the red pulp of the spleen.

**Supplementary Figure 8**

(a) Schematic of the molecular beacon assay for G:T repair; dose dependence curves for APE1 (b) and TDG (c).

**Supplementary Figure 9**

Cell viability of HEMn-LP melanocytes treated with increasing doses of juglone and closantel for 72hrs (a) and 96hrs (b).
