# Supplementary figures and images for: Thymine DNA glycosylase as a novel target for melanoma
Source: Oncogene. 2019 Jan 23;38(19):3710–28. doi: 10.1038/s41388-018-0640-2 (PMC6563616; doi:10.1038/s41388-018-0640-2)

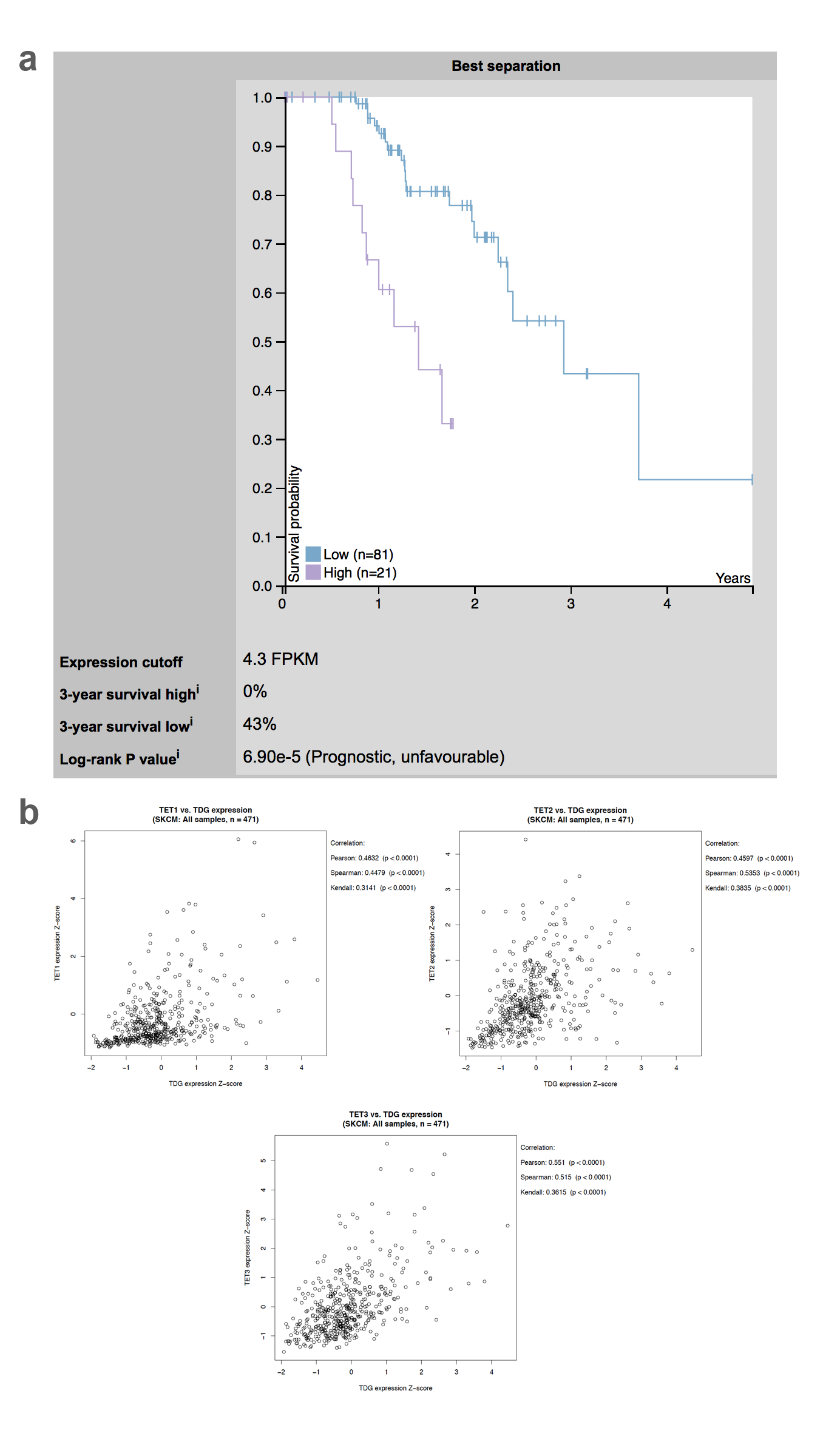

Supplement: Supplementary file 2 — Supplementary Figure 1 [file 41388_2018_640_MOESM2_ESM.tif]

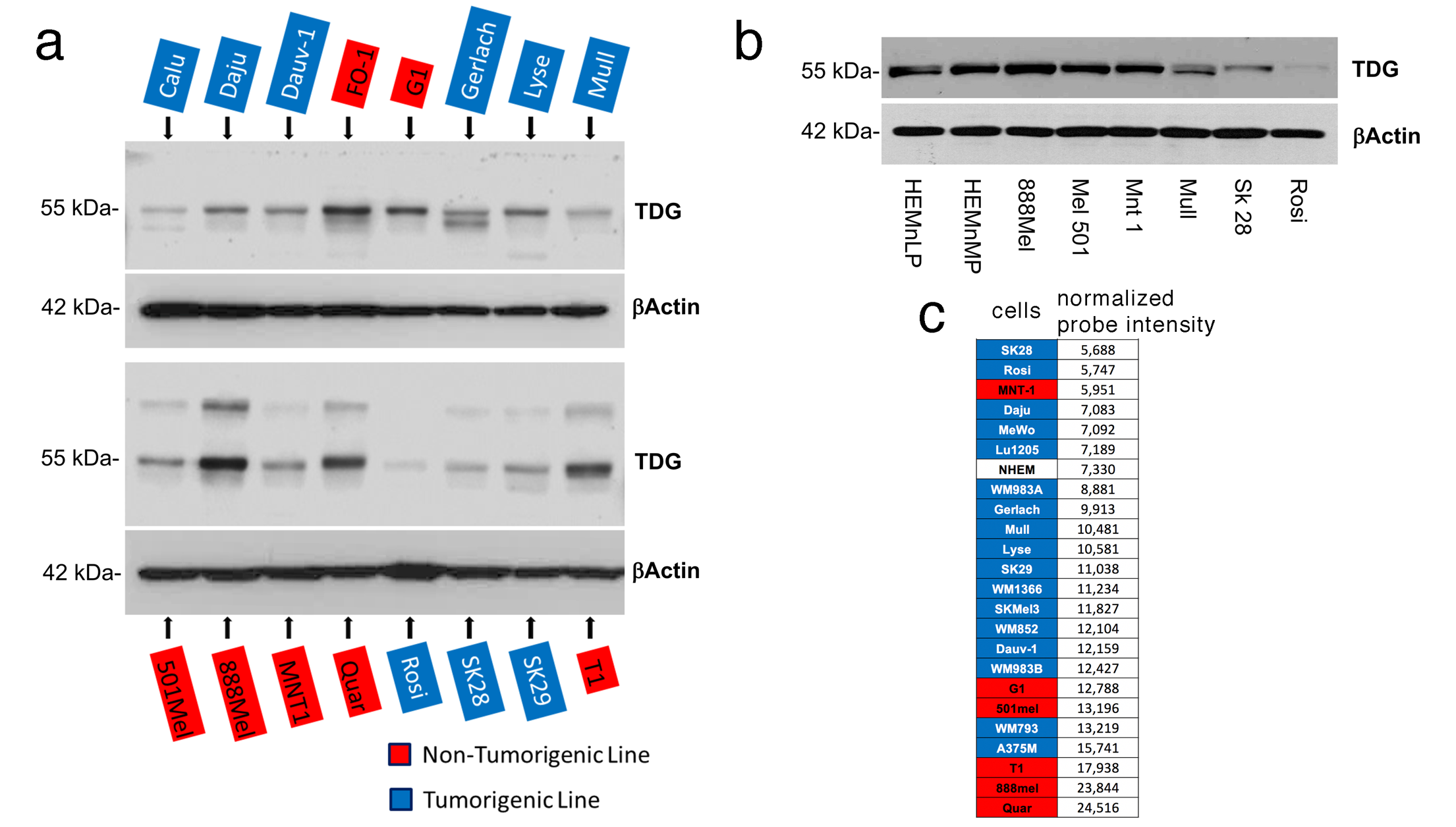

Supplement: Supplementary file 3 — Supplementary Figure 2 [file 41388_2018_640_MOESM3_ESM.tif]

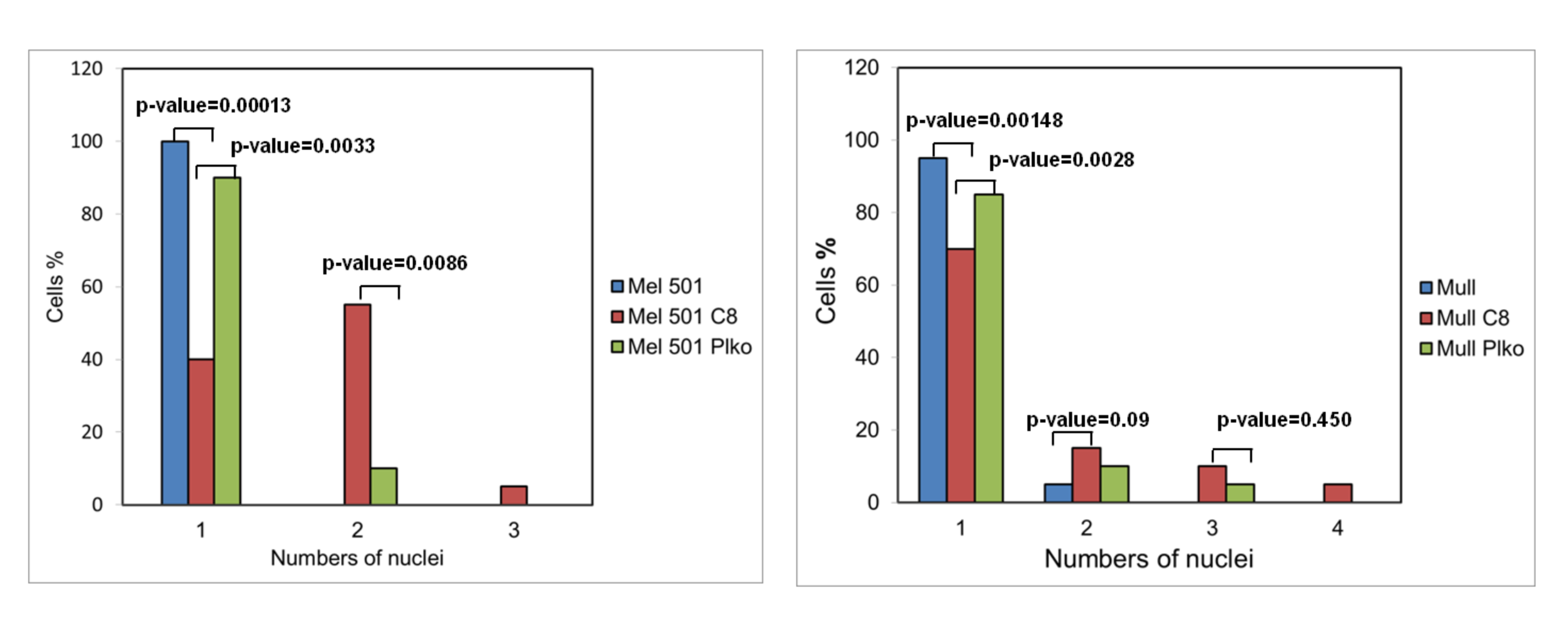

Supplement: Supplementary file 4 — Supplementary Figure 3 [file 41388_2018_640_MOESM4_ESM.tif]

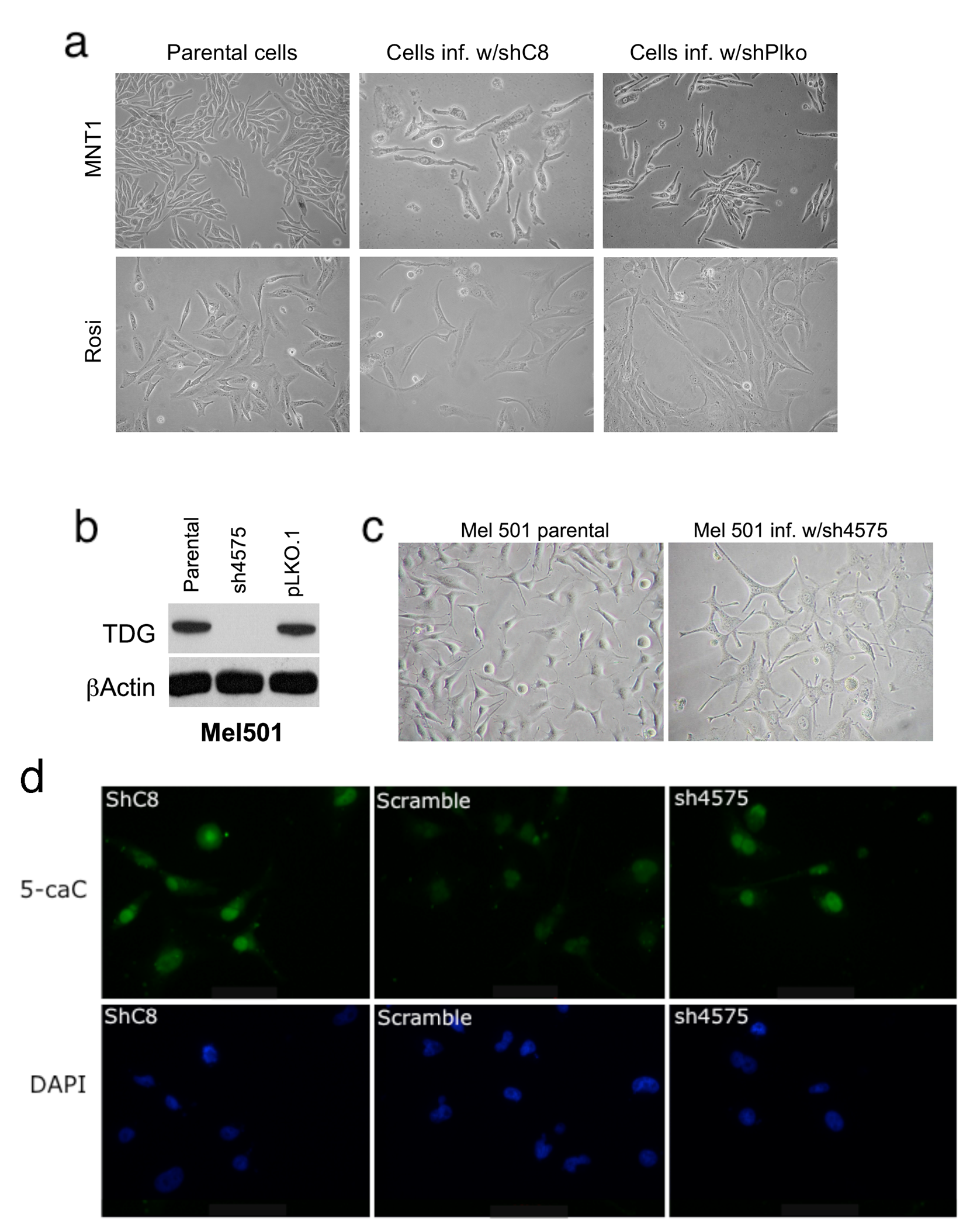

Supplement: Supplementary file 5 — Supplementary Figure 4 [file 41388_2018_640_MOESM5_ESM.tif]

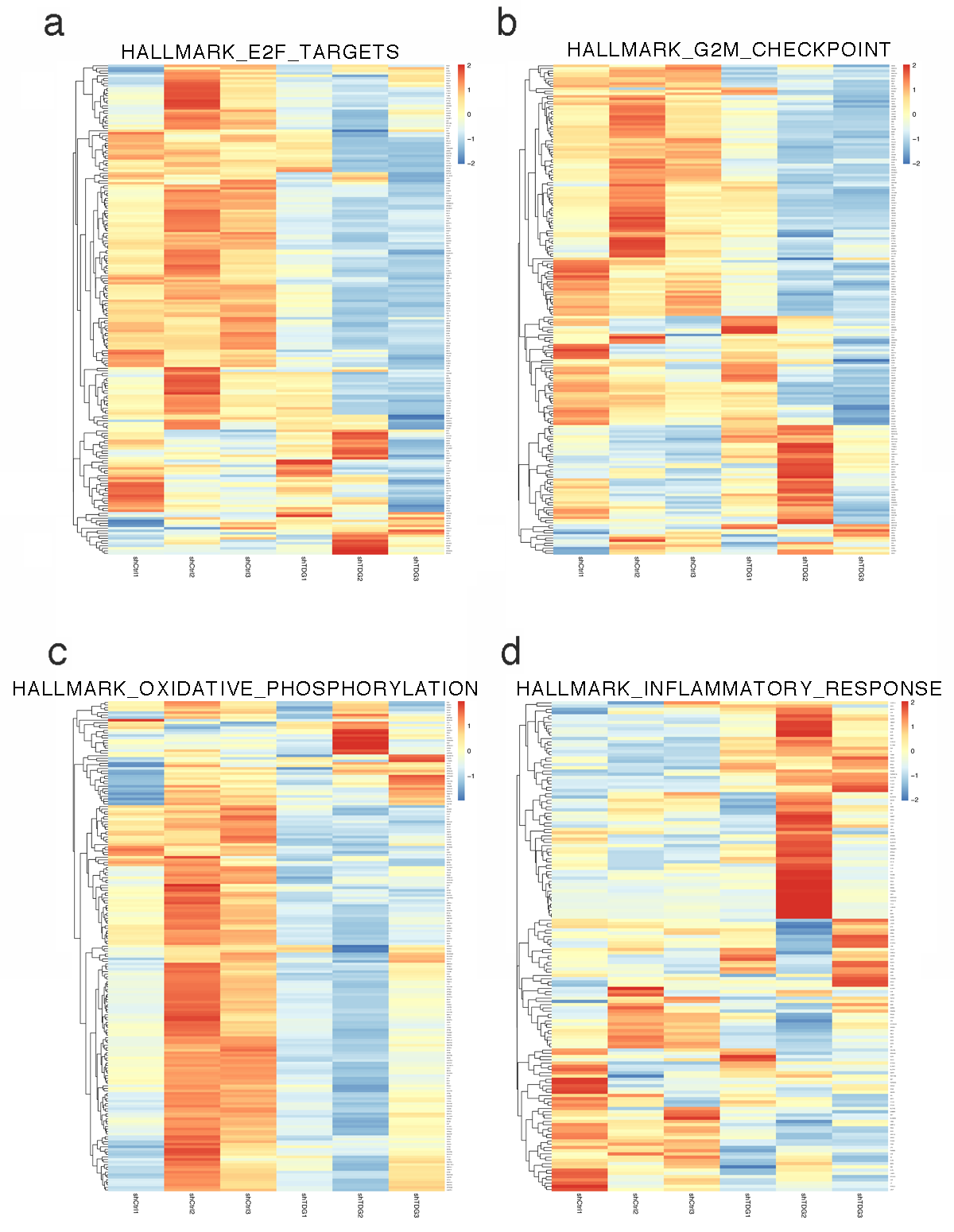

Supplement: Supplementary file 6 — Supplementary Figure 5 [file 41388_2018_640_MOESM6_ESM.tif]

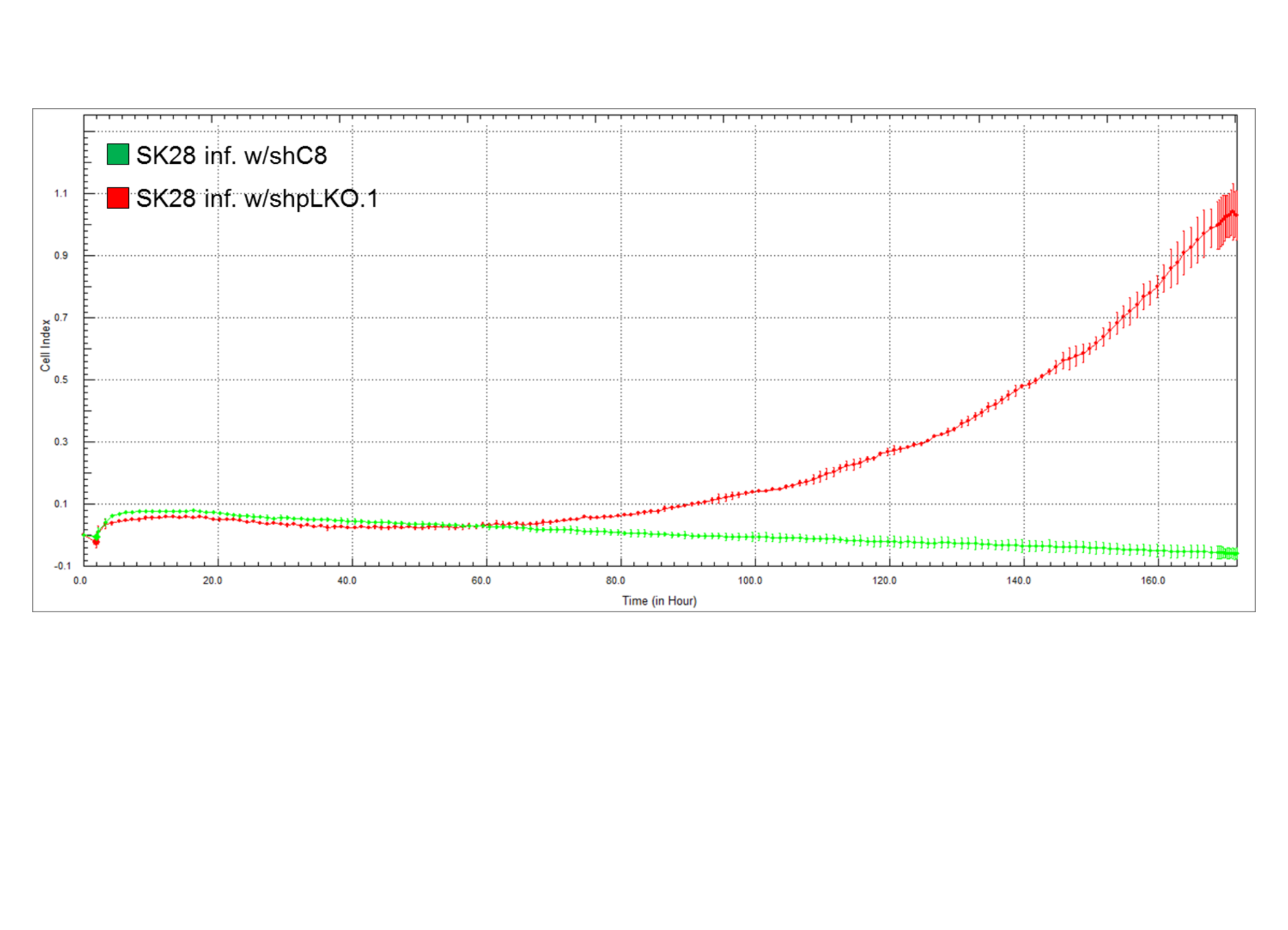

Supplement: Supplementary file 7 — Supplementary Figure 6 [file 41388_2018_640_MOESM7_ESM.tif]

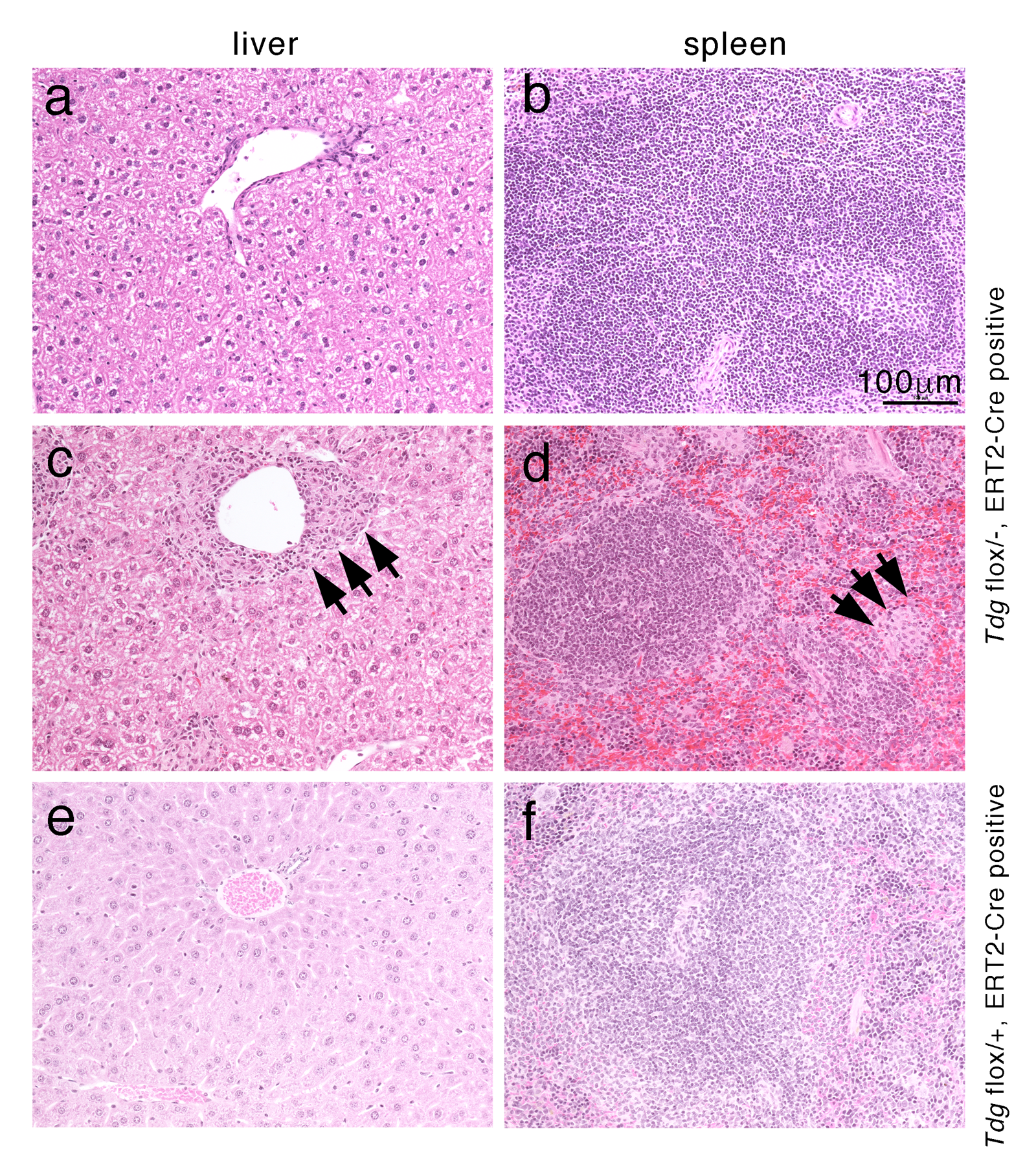

Supplement: Supplementary file 8 — Supplementary Figure 7 [file 41388_2018_640_MOESM8_ESM.tif]

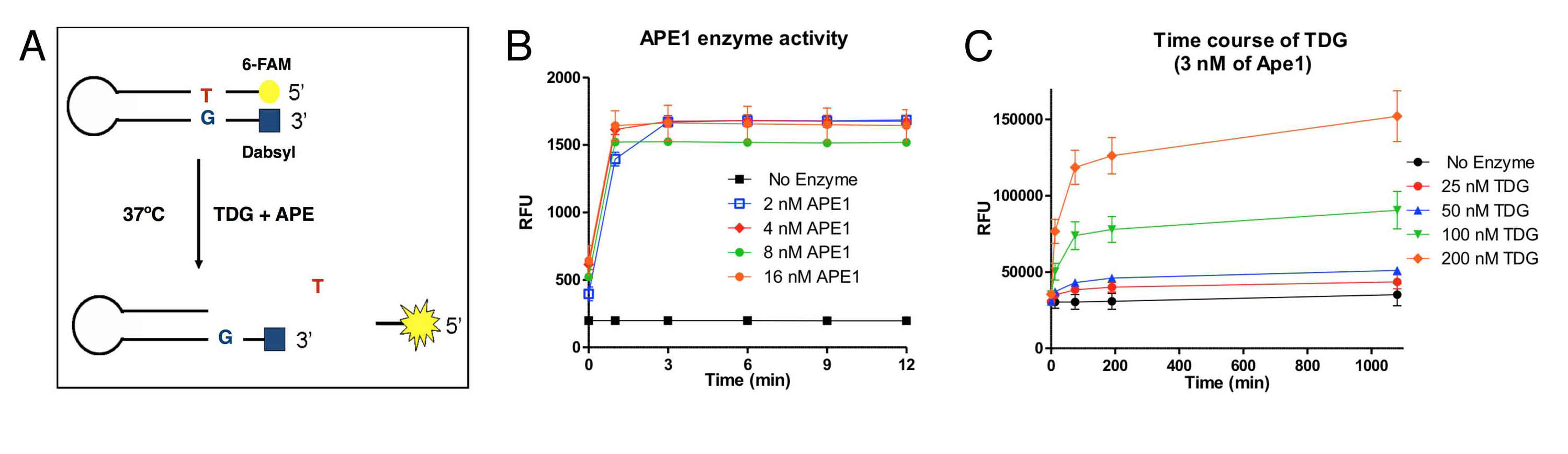

Supplement: Supplementary file 9 — Supplementary Figure 8 [file 41388_2018_640_MOESM9_ESM.tif]

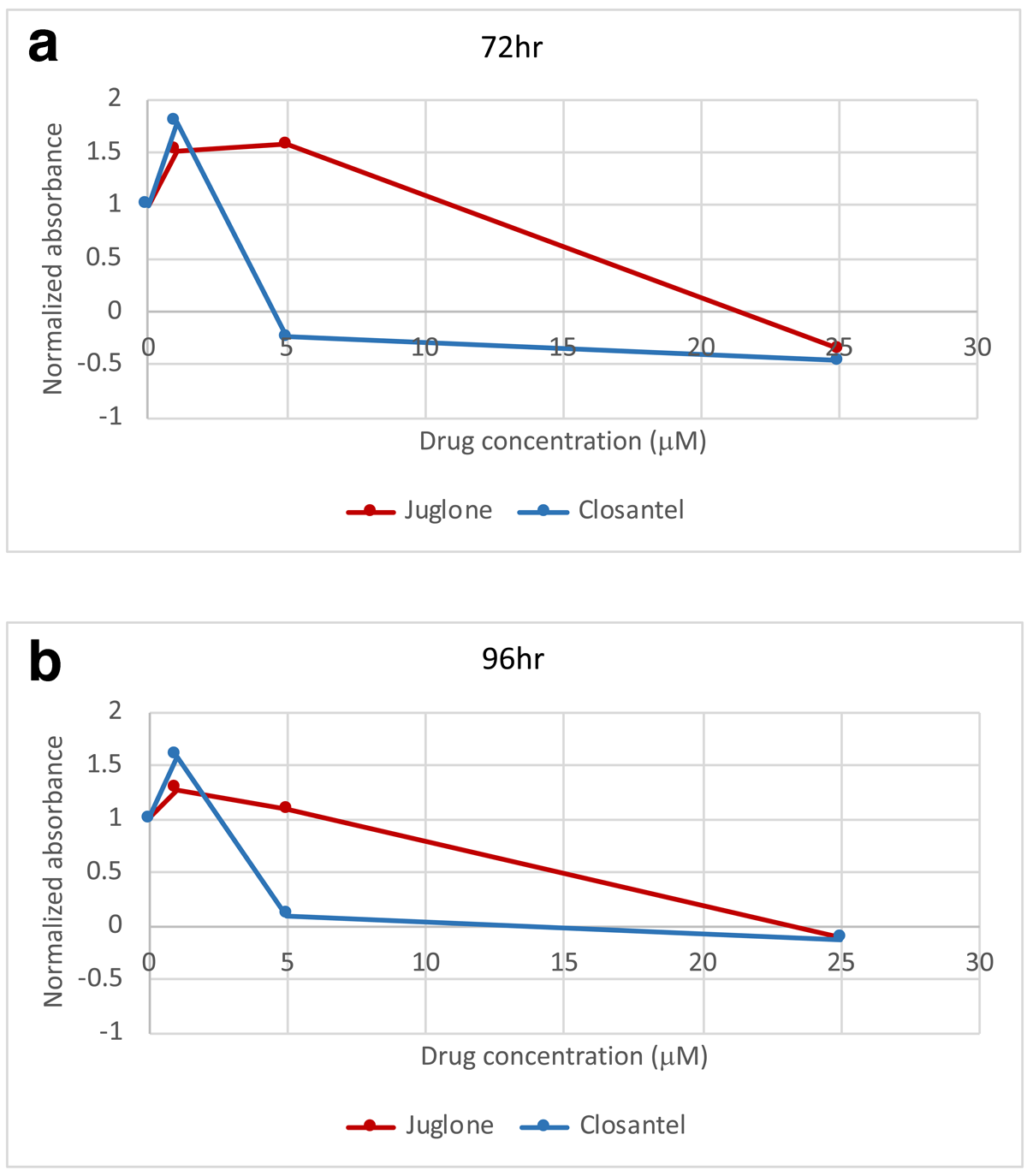

Supplement: Supplementary file 10 — Supplementary Figure 9 [file 41388_2018_640_MOESM10_ESM.tif]
