# Supplementary material for: Thymine DNA glycosylase as a novel target for melanoma
Source: Oncogene. 2019 Jan 23;38(19):3710–28. doi: 10.1038/s41388-018-0640-2 (PMC6563616; doi:10.1038/s41388-018-0640-2)
Supplement: Supplementary file 11 — Supplementary Tables 1-2-3 [file 41388_2018_640_MOESM11_ESM.docx]

Supplemental Table 1. Candidate TDG inhibitors identified by screening the ICCB library.

| 6-Keto-prostaglandin F1a  7-[(1R,2S)-2-[(E,3S)-3-hydroxyoct-1-enyl]-5-oxocyclopent-3-en-1-yl]heptanoic acid | 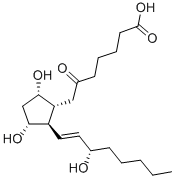 |
| --- | --- |
| E6 Berbamine  6,6',7-Trimethoxy-2,2'-dimethylberbaman-12-yl acetate | 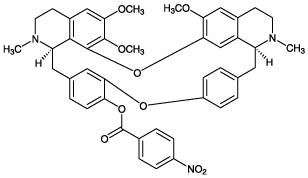 |
| Prostaglandin A1  9-oxo-15S-hydroxy-prosta-10,13E-dien-1-oic acid | [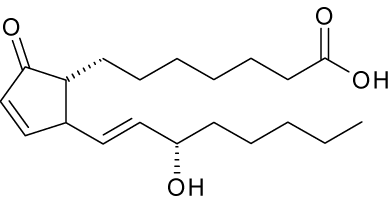](http://upload.wikimedia.org/wikipedia/commons/e/e5/Prostaglandin_A1.png) |
| Juglone  5-hydroxynaphthoquinone | [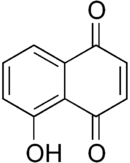](http://en.wikipedia.org/wiki/File:Juglone.png) |
| GW-5074  3-(3,5-Dibromo-4-hydroxybenzylidine-5-iodo-1,3-dihydro-indol-2-one | 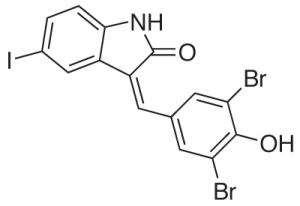 |
| Rottlerin  (E)-1-[6-[(3-acetyl-2,4,6-trihydroxy-5-methylphenyl)methyl]-5,7-dihydroxy-2,2-dimethylchromen-8-yl]-3-phenylprop-2-en-1-one | 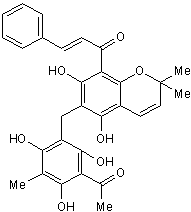 |

Supplemental Table 2. Candidate TDG inhibitors identified by screening the JHCC library.

| Cefixime  (6R,7R)-7-{[2-(2-amino-1,3-thiazol-4-yl)-2-(carboxymethoxyimino)acetyl]amino}-3-ethenyl-8-oxo-5-thia-1-azabicyclo[4.2.0]oct-2-ene-2-carboxylic acid | [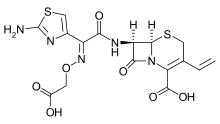](http://en.wikipedia.org/wiki/File:Cefixime.svg) |
| --- | --- |
| Idarubicin  (1S,3S)-3-acetyl-3,5,12-trihydroxy-6,11-dioxo-1,2,3,4,6,11-hexahydrotetracen-1-yl 3-amino-2,3,6-trideoxo-α-L-lyxo-hexopyranoside | [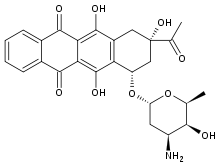](http://en.wikipedia.org/wiki/File:Idarubicin.svg) |
| Doxorubicin  (7S,9S)-7-[(2R,4S,5S,6S)-4-amino-5-hydroxy-6-methyloxan-2-yl]oxy-6,9,11-trihydroxy-9-(2-hydroxyacetyl)-4-methoxy-8,10-dihydro-7H-tetracene-5,12-dione | 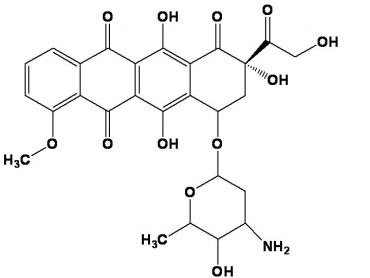 |
| Methenamine (Hexamethylenetetramine) | 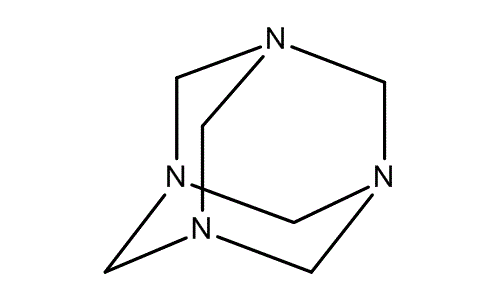 |
| Congo red  disodium 4-amino-3-[4-[4-(1-amino-4-sulfonato-naphthalen-2-yl)diazenylphenyl]phenyl]diazenyl-naphthalene-1-sulfonate | [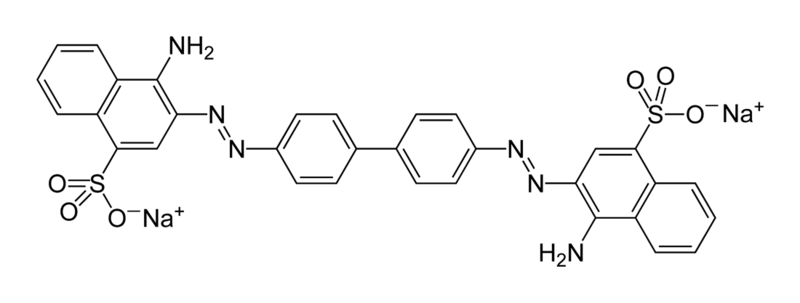](http://upload.wikimedia.org/wikipedia/commons/c/c9/Congo-red-2D-skeletal.png) |
| Sodium ferric gluconate (Ferrlecit®) | 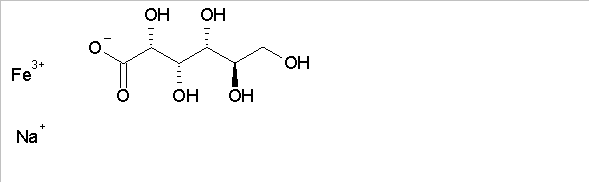 |
| Ferrous sulfate | [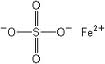](https://www.google.com/imgres?imgurl&imgrefurl=http://www.alanwood.net/pesticides/ferrous%20sulfate.html&h=0&w=0&tbnid=_4vi0l82nHbfGM&zoom=1&tbnh=64&tbnw=105&docid=ORR_H47ErYJkTM&hl=en&tbm=isch&ei=bKaEU8XwHcvKsQTa_YKAAg&ved=0CAUQsCUoAQ) |
| Aurothioglucose  gold(I) (2S,3S,4R,5S)-3,4,5-trihydroxy-6-(hydroxymethyl)-oxane-2-thiolate | [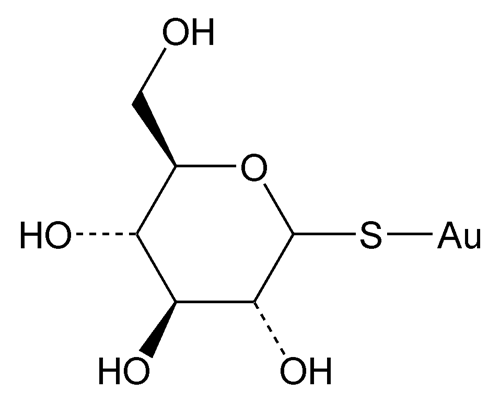](http://www.drugfuture.com/Pharmacopoeia/USP32/pub/data/images/v32270/cas-12192-57-3.gif) |
| Evans blue  tetrasodium (6E,6'E)-6,6-[(3,3'-dimethylbiphenyl-4,4'-diyl)di(1E)hydrazin-2-yl-1-ylidene]bis(4-amino-5-oxo-5,6-dihydronaphthalene-1,3-disulfonate) | 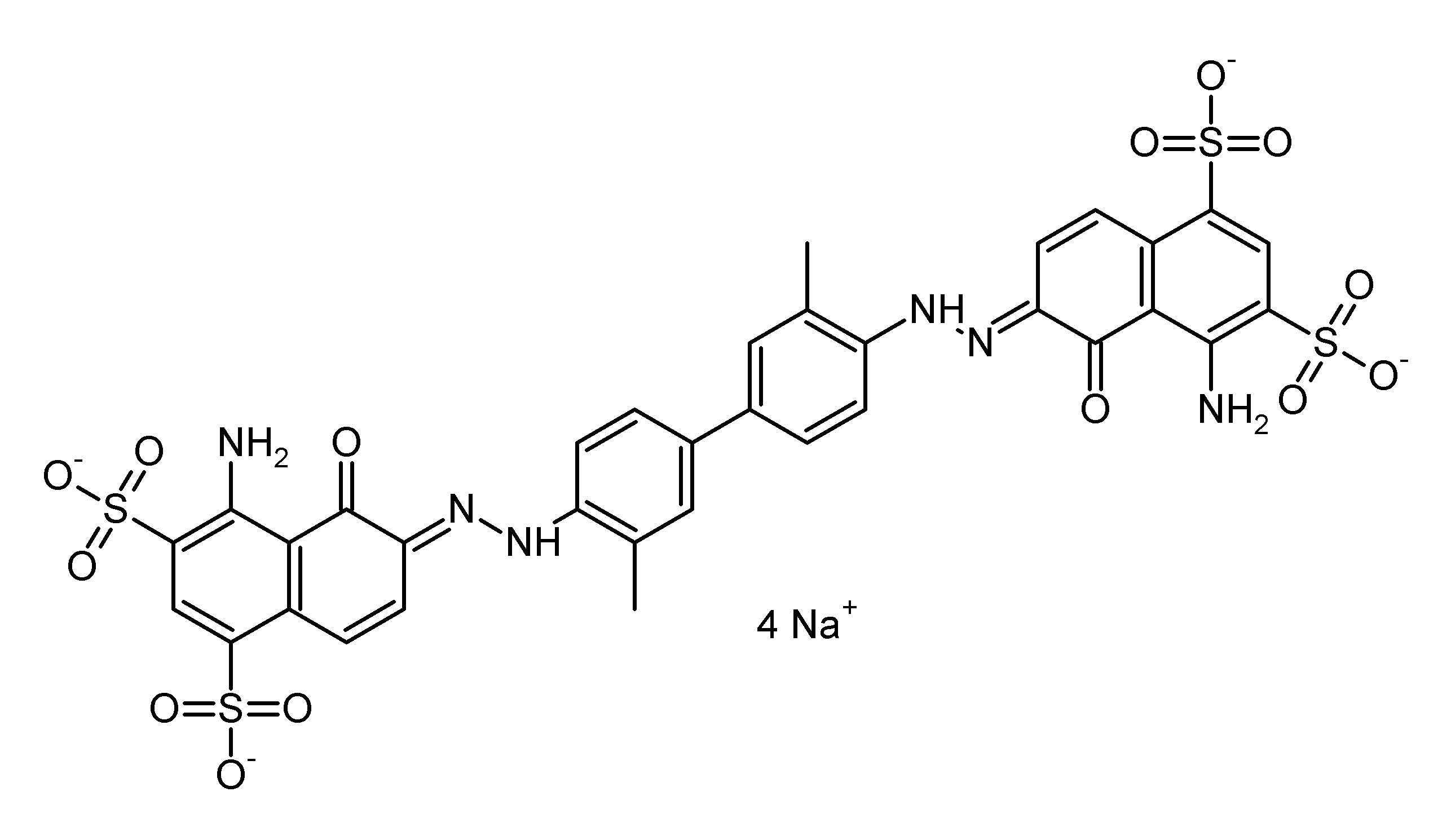 |
| Closantel  5′-Chloro-4′-(4-chloro-α-cyanobenzyl)-3,5-diiodo-2′-methylsalicylanilide, N-[5-Chloro-4-(4-chloro-α-cyanobenzyl)-2-methylphenyl]-2-hydroxy-3,5-diiodobenzamide | 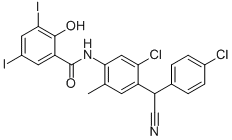 |
| Cinchonine sulfate | [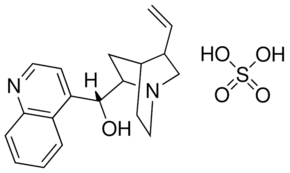](http://www.sigmaaldrich.com/catalog/product/aldrich/s654752?lang=en&region=US) |
| Hexadimethrine bromide (Polybrene)  1,5-dimethyl-1,5-diazaundecamethylene  polymethobromide, hexadimethrine bromide - | [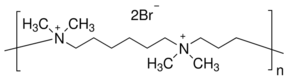](http://www.sigmaaldrich.com/catalog/product/sigma/h9268?lang=en&region=US) |
| Indigotindisulfonate  (Indigo Carmine)  3,3'-dioxo-2,2'-bis-indolyden-5,5'-disulfonic acid disodium salt | [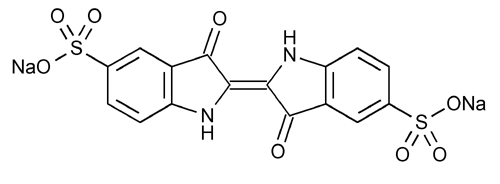](http://www.google.com/url?sa=i&rct=j&q=&esrc=s&frm=1&source=images&cd=&cad=rja&uact=8&docid=2dYsZPkGu8e2cM&tbnid=NfdKub_iIw3ioM:&ved=0CAUQjRw&url=http://www.pharmacopeia.cn/v29240/usp29nf24s0_m40140.html&ei=R6iEU9DLHeHisATAz4GwCw&bvm=bv.67720277,d.aWw&psig=AFQjCNE4JGfoQYtWpwoEkMMlORhFS3UOrA&ust=1401289135842618) |
| Protamine chloride, grade V | MPRRRRSSSRPVRRRRRPRVSRRRRRRGGRRRR |

Supplemental Table 3. Candidate TDG inhibitors identified by screening the LOPAC library.

| (-)-Gossypol  7-(8-formyl-1,6,7-trihydroxy-3-methyl-5-propan-2-ylnaphthalen-2-yl)-2,3,8-trihydroxy-6-methyl-4-propan-2-ylnaphthalene-1-carbaldehyde | 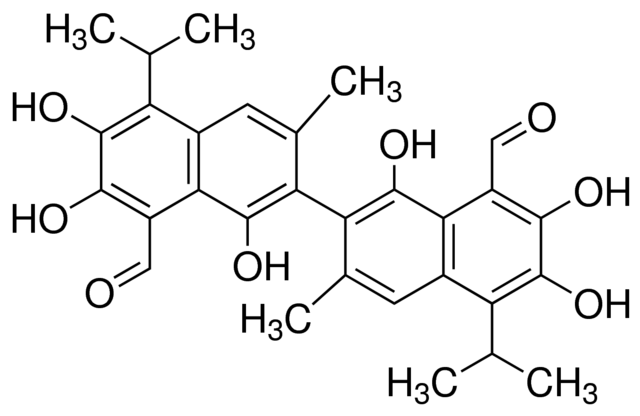 |
| --- | --- |
| NF 023  hexasodium;8-[[3-[[3-[(4,6,8-trisulfonatonaphthalen-1-yl)carbamoyl]phenyl]carbamoylamino]benzoyl]amino]naphthalene-1,3,5-trisulfonate | 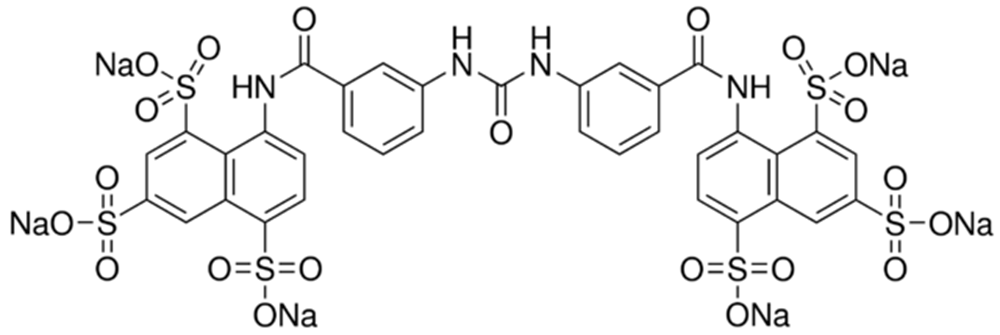 |
| Mitoxantrone  1,4-dihydroxy-5,8-bis[2-(2-hydroxyethylamino)ethylamino]anthracene-9,10-dione | 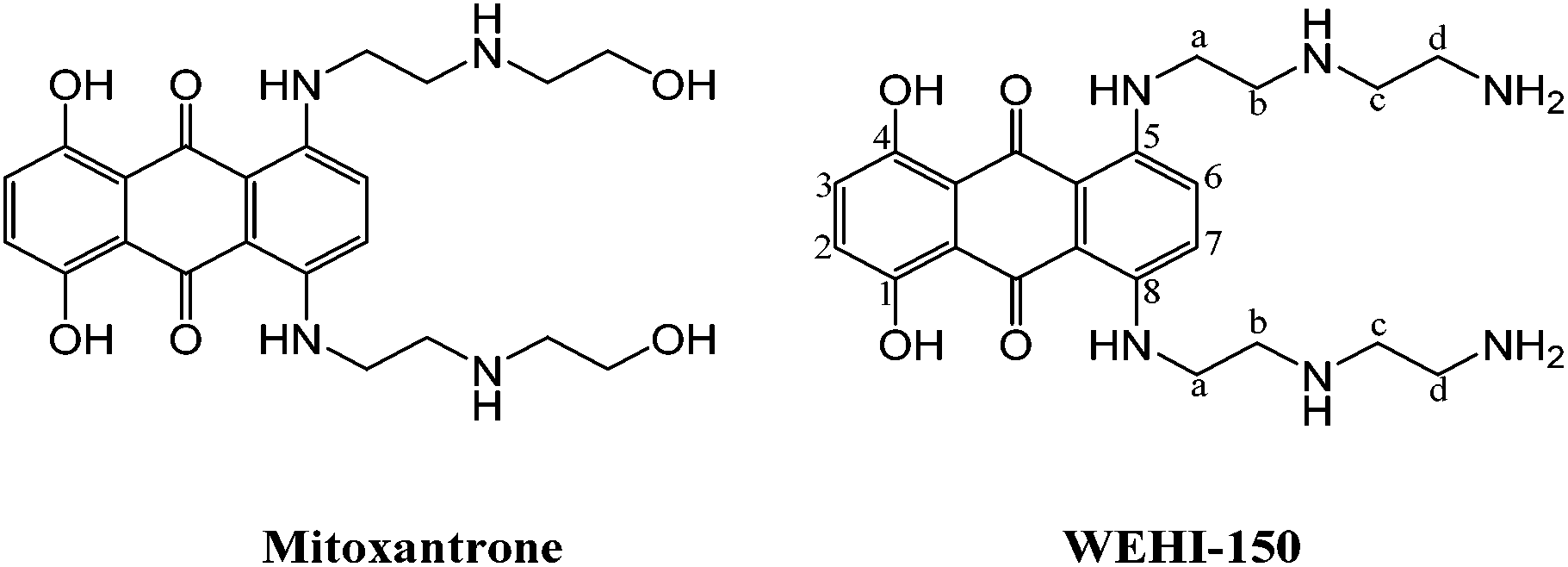 |
| Ro 90-7501  2-[2-(4-aminophenyl)-3H-benzimidazol-5-yl]-3H-benzimidazol-5-amine | 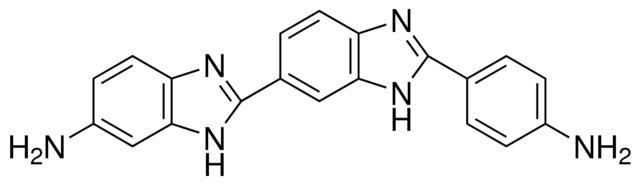 |
| Aurothioglucose  gold(1+);(2R,3R,4S,5S,6R)-3,4,5-trihydroxy-6-(hydroxymethyl)oxane-2-thiolate | 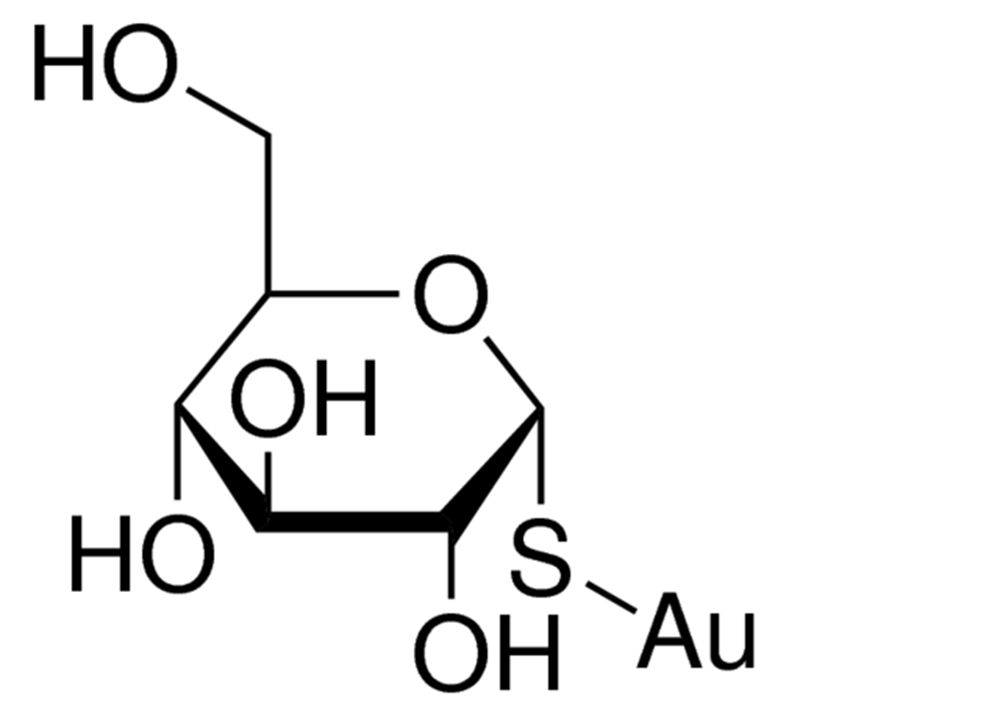 |
| Ethopropazine hydrochloride  N,N-diethyl-1-phenothiazin-10-ylpropan-2-amine;hydrochloride | 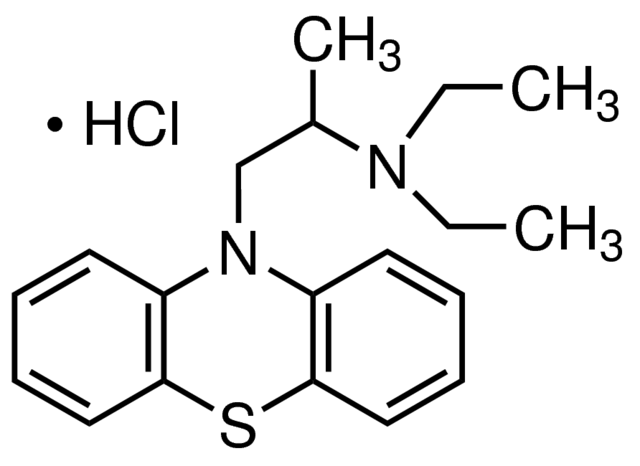 |
| Suramin  8-[[4-methyl-3-[[3-[[3-[[2-methyl-5-[(4,6,8-trisulfonaphthalen-1-yl)carbamoyl]phenyl]carbamoyl]phenyl]carbamoylamino]benzoyl]amino]benzoyl]amino]naphthalene-1,3,5-trisulfonic acid | 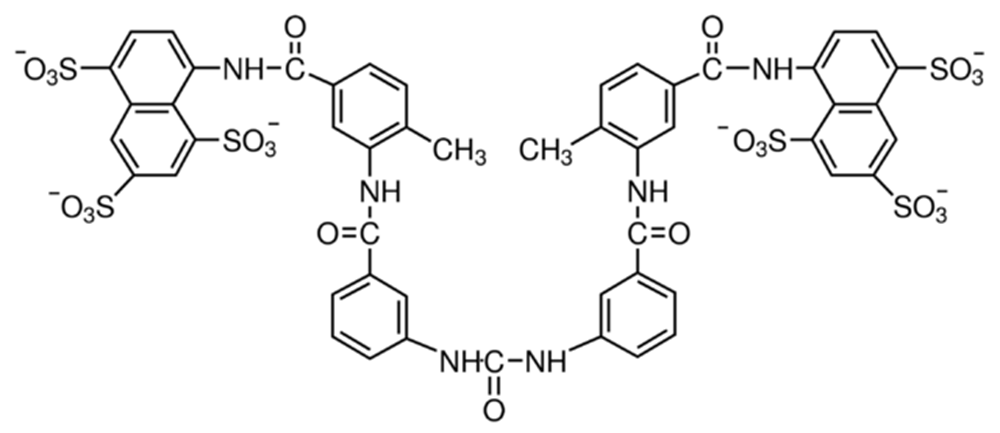 |
| IPA-3  1-[(2-hydroxynaphthalen-1-yl)disulfanyl]naphthalen-2-ol | 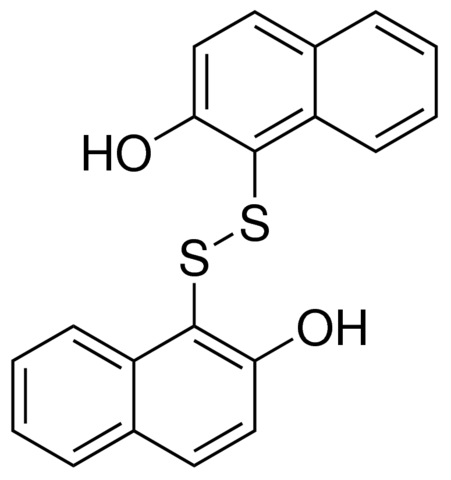 |
| Oxotrernorine methiodide  trimethyl-[4-(2-oxopyrrolidin-1-yl)but-2-ynyl]azanium;iodide | 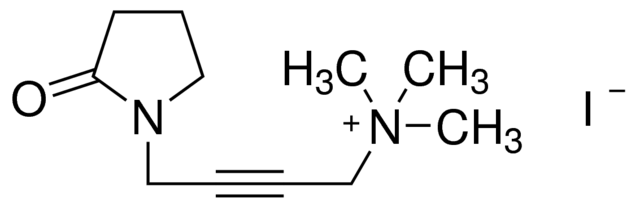 |
